# Flexible reaction norms to environmental variables along the migration route and the significance of stopover duration for total speed of migration in a songbird migrant

**DOI:** 10.1186/s12983-017-0203-3

**Published:** 2017-03-20

**Authors:** Heiko Schmaljohann, Simeon Lisovski, Franz Bairlein

**Affiliations:** 1 Institute of Avian Research “Vogelwarte Helgoland”, An der Vogelwarte 21, Wilhelmshaven, 26836 Germany; 2 University of Alaska, Fairbanks, AK USA; 3 Centre for Integrative Ecology, Deakin University, Geelong, VIC 3220 Australia; 4 Department of Neurobiology, Physiology, and Behavior, University of California, Davis, CA 95616 USA

**Keywords:** Anthropogenic changes, Bird migration, Departure probability, Environmental change, Phenotypic response, Reaction norm, Route, Speed, Travel speed

## Abstract

**Background:**

Predicting the consequences of continuing anthropogenic changes in the environment for migratory behaviours such as phenology remains a major challenge. Predictions remain particularly difficult, because our knowledge is based on studies from single-snapshot observations at specific stopover sites along birds’ migration routes. However, a general understanding on how birds react to prevailing environmental conditions, e.g. their ‘phenotypic reaction norm’, throughout the annual cycle and along their entire migration routes is required to fully understand how migratory birds respond to rapid environmental change.

**Results:**

Here, we provide direct evidence that northern wheatears (*Oenanthe oenanthe*) from a breeding population in Alaska adjusted their probability to resume migration as well as the distance covered per night, i.e. travel speed, to large-scale environmental conditions experienced along their 15,000 km migratory route on both northwards and southwards migrations. These adjustments were found to be flexible in space and time. At the beginning of autumn migration, northern wheatears showed high departure probabilities and high travel speeds at low surface air temperatures, while far away from Alaska both traits decreased with increasing air temperatures. In spring, northern wheatears increasingly exploited flow assistance with season, which is likely a behavioural adjustment to speed up migration by increasing the distance travelled per night. Furthermore, the variation in total stopover duration but not in travel speed had a significant effect on the total speed of migration, indicating the prime importance of total stopover duration in the overall phenology of bird migration.

**Conclusion:**

Northern wheatears from Alaska provide evidence that the phenotypic reaction norm to a set of environmental conditions cannot be generalized to universal and persistent behavioural reaction pattern across entire migratory pathways. This highlights the importance of full annual-cycle studies on migratory birds to better understand their response to the environment. Understanding the mechanisms behind phenotypic plasticity during migration is particularly important in the assessment of whether birds can keep pace with the potentially increasing phenological mismatches observed on the breeding grounds.

**Electronic supplementary material:**

The online version of this article (doi:10.1186/s12983-017-0203-3) contains supplementary material, which is available to authorized users.

## Background

Human-induced climate change [1] has altered temporal patterns in the environment [2], resulting in a general advance in the arrival times of migratory birds to their breeding grounds [3–6]. However, the responses of species to rapidly changing environments are not synchronized which often leads to serious phenological mismatches between trophic levels [7–9]. And although many long-distance migratory bird species have significantly advanced their spring phenology [4, 10, 11], their current worldwide decline [12] indicates remaining or even increasing phenological mismatches with their primary food sources [13–17]; but see [18, 19]. Three non-mutually exclusive proximate causes can explain variation in arrival dates: start of migration, total migration distance, and total speed of migration, i.e. the average migratory distance covered per day including periods of stopover during the entire migratory period [km/day], [20, 21]. Furthermore, ontogenetic factors [5] as well as exogenous phenological events at the breeding area may influence the timing of the various life-history stages in migratory birds [22]. Conditions at the wintering grounds may also be used as a cue to initiate spring migration [23, 24], which is a good predictor for breeding area arrival timing [25–29]. However, high within-individual repeatability in the timing of spring migration in some species [27, 30] suggest that conditions at the wintering grounds may not necessarily influence arrival dates [31, 32]. If migrants experience favourable environmental conditions along the entire migratory route, the arrival at breeding grounds can potentially be adjusted by increasing the total speed of migration [20, 33, 34]. Yet, this adjustment might be limited since the rate of accumulating energy at a stopover is much slower than the rate of spending energy in flight, indicating that stopovers are likely the crucial periods of the migratory journey affecting total speed of migration [35].

To date, our knowledge on the effects of the environment, e.g. prevailing weather conditions, on stopover duration and travel speed, i.e. the daily migratory distance covered during periods of flight, is primarily inferred from single-snapshot studies detailing the behaviour of individuals at a specific site [36, 37] or along a single migratory leg [38–40]. Yet, individuals may react differently to a similar set of environmental conditions [41] depending on e.g. the distance remaining to the destination [42]. Information from one radar-tracking study from various species and two locations suggested that individuals tracked closed to the breeding area had a higher migration speed than those tracked further away [43]. This suggests that the biological significance of environmental parameters may change during migration. In contrast to large species [44–51], it remains difficult to follow individual songbirds over large distances and entire annual cycles [52, 53]. Due to this constraint, we are still restricted in our ability to identify environmental parameters that determine the entire migratory schedule and whether stopover duration significantly affects the travel speed in long-distance migratory songbirds. In this study, we used light-level geolocators to track northern wheatears (*Oenanthe oenanthe*, wheatear hereafter), a small nocturnal migratory songbird, from a population breeding in Alaska. Wheatears are exclusive nocturnal migrants [54] and individuals breeding in Alaska are known to travel approximately 15,000 km from Alaska to their African wintering grounds [55]. Our general aim was to investigate the extent to which birds adjust their departure timing and travel speed in response to local environmental conditions, and whether such decisions are influenced by the proximity of the breeding areas and/wintering grounds.

Our first objective was the quantification of the direct effects of environmental variation on daily departure probability and daily travel speed along the migration route. Using remotely sensed environmental parameters to describe weather changes and conditions along the migration route and within the regional stopover sites, we aim to model how each of these parameters influenced the daily probability of an individual to resuming migration, as well as the travel speed of each migration bout. We hypothesize that the daily departure probability increases with decreasing temperature in autumn [54, 56], as a mechanism to avoid high energy costs at low surface temperature during the night [57]. Furthermore, in spring, we expect birds to slow down their migration or even reverse migration, if the temperature drops significantly due to high energy costs at cold conditions [57]. The likelihood of this process may increase towards the breeding area in the Arctic and with even colder conditions. In addition, we predict that departure probability from stopover sites will increase with decreasing precipitation [36, 58, 59] and high air pressure [60–62]. Lower wind speeds [58, 59, 63] and favourable wind assistance [63, 64] are also known to increase the probability to resume migration and to also affect the individuals’ travel speed [65]. Given the restrictions of nocturnal migrants to gain distance ‘solely’ during the night, early departures would allow birds to extent flight duration and potentially achieve larger distances per night. It is therefore assumed that favourable environmental conditions on the ground would results in early night departures, whereas less favourable conditions may lead to a higher variation in the nocturnal departure time [66]. Finally, during spring migration we expect that prevailing climatic conditions would become less important to decision making the closer, the individual is to the breeding area: timely arrival at the breeding grounds is crucial [67], and individuals might be forced to resume migration, even if conditions are unfavourable once they get closer to the breeding area [42, 43]. Here we will provide evidence whether the phenotypic reaction norm to a set of environmental conditions is fixed or flexible across entire migratory pathways of northern wheatears in both seasons and in accordance with the above described predictions from single snap-shot studies. Our second objective was to quantify the direct effects of stopover duration and travel speed on total speed of migration and estimate the importance for arrival timing at the migratory destination. According to previous findings [68], we expect that seasonal variation in stopover duration of individual weathers and not mean travel speed determines their total speed of migration.

## Methods

### Study site and light-level geolocators

Wheatears were ringed and tagged at two sites in Alaska (Eagle Summit: 65.6° N, 145.4° W and Toolik: 68.6° N, 149.6° W, U.S.A.; Additional file 1). In 2009, 15 adult male and female wheatears were tagged with Mk10S geolocators from British Antarctic Survey (Cambridge, UK; 1.4 g representing max. 6% of bird’s body mass) at Eagle Summit [56]. In 2013, 28 adult male, 32 adult female and 60 juvenile wheatears were tagged at Eagle Summit and 23 adult male and female and 74 juvenile wheatears were tagged at Toolik. In 2013, we used ML6190 geolocators from Biotrack (Wareham, UK; 0.9 g representing max. 4% of bird’s body mass) and Intigeo P65A9-11 from Migrate Technology (Cambridge, UK; 0.9 g). Adult body mass of tagged individuals was 26.0 ± 2.2 g (mean ± s.d., *n* = 135) compared to juveniles with a body mass of 25.9 ± 1.8 g (*n* = 134). Further information on the light-level geolocator devices as well as explanations on recapture probabilities can be found in Additional files 1 and 2.

### Analyses of light-level geolocation data

Light-level geolocators archive light intensity over time. For any given site, light intensity changes specifically over the year with respect to a standard time, allowing estimation of positions [69, 70]. The uncertainty of those estimates is frequently described to be within 100–200 km, but is highly dependent on mainly the bird’s behaviour which affects the shading of the light sensor, and the time of the year [71]. In addition, the accuracy and precision of the estimates depends on the analytical approach, see [72–74].

In this study, light-level geolocator data, available upon request from “Movebank” (www.movebank.org, project: *Oenanthe oenanthe* Alaska), were analysed using the software R 3.1.2 (Vienna, Austria) [75] with the freely available R packages “SGAT” [76] and “BAStag” [77], cf. [74, 78]. First, daily sunrise and sunset times were identified using the arbitrary light intensity of “1.35” for the BAS and Biotrack geolocators and 1.35 lux for the Intigeos, similar as in other studies [74, 78, 79] (Additional file 1). Ninety-three (0.02%) out of 4,606 sunrise and sunset times were discarded and 99 (0.02%) of the remaining ones were the difference was > 30 min from the otherwise stable surrounding twilight events were adjusted (Additional files 1 and 3). Initial locations were estimated using the simple threshold method with a zenith angle of 96.8 ± 1.3° (mean ± s.d.; *n* = 8). Next, a Bayesian approach was used to refine the simple threshold estimates and describe the posterior distribution of bird locations [73]. Within a Markov Chain Monte Carlo simulation we incorporated a priori knowledge in terms of a species-specific land mask, a twilight model, and a species-specific movement model to obtain a likelihood surface for location estimates [73]; see [74] for further details. The land mask biased potential locations towards land, given that the wheatear is a typical land bird [80]; thus, the relative probability to be over land was higher than that to be over water. This mask was applied for daytime and night-time location estimates. The twilight model described the expected error in the detection of sunrise and sunset times. Since natural light cannot be detected before sunrise or after sunset, we used a log-normal density distribution with the parameters meanlog = 2.2 and sdlog = 1.0 [74]. Parameters for the log-normal distribution were estimated using light intensity recordings from the breeding site (known location) pooled across all individuals. The zenith angle was then estimated for each individual separately. Songbirds are known to spend most of their time during migration at stopover sites [56, 57, 81]. The movement model has been parameterised to account for high probabilities of low speeds and lower probability of high speeds by assuming a gamma distribution with shape = 0.7 and scale = 0.05. The expected mean airspeed of wheatear’s is approximately 50 km/h [82]. For further information about the movement model see Additional file 3. Parameters of these three models were used for each individual to describe the posterior distribution of estimated locations. Additional file 3 provides the corresponding R code.

### Defining migration schedule

To discriminate between stopovers and periods of migratory flights, we used the “changeLight” function of the R package “GeoLight” [83]. This method is based on a “changepoint” analysis that quantifies the probability of each sunrise and sunset to be different than the surrounding sunrise and sunset times and provided evidence for shifts/movement in the underlying locations. The sunrise and sunset times associated with a “changepoint” probability greater than the 0.75 quantile of all probabilities were used to define stationary periods separated by periods of movement. Using the 0.75 quantile of all “changepoint” probabilities and only considering stopovers of two days or longer is a conservative approach [83]. If the mean of the location estimates of consecutive stationary periods fall within a radius of 200 km, we merged those considered sites to be one. However, merging of stationary periods occurred only once in two birds at the beginning of autumn migration. By definition, birds resumed migration on the last evening of a stopover. Because the selected zenith influenced latitude estimates, the onset of autumn migration was defined as the start of the period of consecutive migratory flights preceding the last stationary site at > 2° of longitude westward of the breeding area. For stopover sites closer than 2° longitude, the last day of the subsequent period of consecutive migratory flights was defined as the onset of autumn migration. Termination of autumn migration was defined by the beginning of the first stationary site in eastern Africa. The onset of spring migration was defined as the first date of the period of consecutive migratory flights preceding the first stationary period 200 km away from the wintering ground. In spring, the last day of the last period of consecutive migratory flights before reaching the breeding area was considered as the termination of spring migration. However, this date could be determined only for one wheatear because for the others, the light intensity was above the threshold of 1.35 for detecting twilight events (see above) when Alaska was reached. Total speed of migration [km/day] was estimated as the total migration distance [km], as tracked by light-level geolocation data, divided by the duration [days] of the entire migration. In spring, total speed of migration was simplified as the distance from bird’s wintering ground to its last location estimate divided by the duration for this subset of the journey. The travel speed [km/days] was defined as the great circle distance [km] covered between daily locations on travel days [day], excluding days of stopover.

### Environmental data

National Centres for Environmental Prediction Reanalysis II data from the National Oceanic and Atmospheric Administration (NOAA, Boulder, CO, USA; [84]) were obtained via the R package ”RNCEP” [85]. This dataset consists of various climatic measurements with a 2.5x2.5° spatial resolution corresponding to ground surface area cells of 120 × 120 km^2^ and 290 × 290 km^2^ for the study area. This area size reflects the general uncertainty of location estimates derived from light-level geolocation; resolution of the weather data and the accuracy and precision of the light-level geolocator data were therefore in the same order of magnitude (see above). Several studies have shown that RNCEP wind data correlated highly significantly with real wind measurements in terms of speed and direction [86–89]. To describe the general environmental conditions that has most likely been experienced by the birds, we downloaded daily surface air temperatures [°C], surface air pressure levels [hPa], and surface wind speed [m/s] for the time of local sunsets for each day of the migration period [84, 85]. The amount of precipitation [kg/m^2^/s] was summed from three hours before until three hours after local sunset. Environmental values were interpolated in space and time [85]. Wheatears perform nocturnal exploratory flights [86] possibly to assess wind conditions aloft [65]. The best flow assistance of four different pressure levels was considered (1000, 925, 850, and 700 mbar) [65, 87, 90, 91]. Flow assistance was quantified using equation^Airspeed^:

$$ flow\  assistance\ \left[\frac{m}{s}\right]= flow\  speed\ \left[\frac{m}{s}\right]\ast \cos \left(\theta \right)+\sqrt{z^2-{\left( y\ast \sin \left(\theta \right)\right)}^2}- z $$

with θ is the angular difference between the direction into which the wind is blowing and the bird’s preferred direction of migratory movement, z is bird’s speed [m/s] relative to the wind, and y is wind speed [m/s], following [92], by applying the “NCEP.flight” function of the R package ”RNCEP” [85], with “flow.assist” set to “NCEP.Airspeed”, “air speed” set to 13 m/s following Bruderer and Boldt [82], and “path” to “great circle”. Thus, the best flow assistance of the four pressure levels to the next migratory goal was considered for each day, cf. [87]. On 48 of the 1038 “bird-days” during both autumn and spring migration, equation^Airspeed^ did not provide an estimate because the wind speed was higher than the assumed airspeed. We assumed that birds perceived these modelled environmental conditions en route and considered these for their daily departure decision. For modelling travel speed the same environment conditions were incorporated. However, flow assistance was modelled for each hour and corresponding wind conditions were interpolated in space and time and separately for the different pressure levels. Thus, birds did not experience an abrupt change in wind conditions when crossing two grid cells.

### Statistical analyses

Statistics were calculated using the R 3.1.2 statistical software package [75]. All dates and times are given in Greenwich Mean Time.

Numeric explanatory variables were z-transformed to achieve homogeneity of variance. All numeric explanatory variables considered in a model were tested against one another for collinearity with the “vif” function of the R package “usdm” [93]. If collinearity exceeded the conservative threshold of 2, one of the corresponding covariates was removed. For values that were lower than 2, the variables were treated as not collinear [94]. Autumn and spring migrations were modelled separately.

The variation in the individual departure probability along the migration route was modelled using a mixed effect logistic regression model (MELR) [95]. Variation in the individual departure probability was related to the environmental conditions at sunset (surface air temperature, precipitation, surface air pressure, surface wind speed, flow assistance, and remaining migratory distance, for details see “Environmental data”). Year of deployment was included as a random factor. Scale parameters ranged between 0.75 and 1.4 (1.06 in the autumn model and 0.98 in the spring model) indicating that overdispersion was not a confounding factor [96]. Furthermore, corresponding goodness of fit plots showed no violation of model assumptions (Additional file 4).

The variation in the travel speed was modelled using a linear mixed effect model (LMM) assuming normally distributed errors [95, 97]. To avoid violation of model assumptions stopover duration was log10-transformed [97]. Surface air temperature, precipitation, surface air pressure, surface wind speed (all sunset values), flow assistance, and remaining migration distance were used as explanatory variables. In this model, the flow assistance was taken as the mean of the hourly flow assistance experienced by the bird towards the next estimated location. Year was included as a random effect. To account for the inherent uncertainty of single location estimates derived from light-level geolocation [71], the data values were weighted by the inverse of the standard deviation of the latitude estimate.

The surface air temperature was collinear with the remaining migration distance in all spring models. Hence, the potential effect of the remaining migration distance on the variation in departure probability and travel speed could not be separated from the potential effect of surface air temperature in the spring models. As a result, surface air temperature was removed from all spring models.

The variation in the total speed of migration was modelled by total stopover duration and season using a LMM and assuming normally distributed errors. The variation in the total speed of migration was also separately modelled by the mean travel speed and season again using a LMM [95, 97]. For both models, individual was included as a random factor and all numeric parameters, i.e. the response and the predictor variables, were log_10_-transformed to avoid violation of model assumptions [97] which we assessed graphically (Additional file 4).

We used improper prior distributions, namely *p(β) ~ 1* for the coefficients, and *p(β) ~ 1/σ* for the variance parameters following [96], which were applied to all models described above. To obtain the posterior distribution, we directly simulated 2,000 values from the joint posterior distribution of the model parameters using the function sim of the package “arm” [98]. The median of the simulated values from the joint posterior distributions of the model parameters was used as an estimate, with the 2.5 and 97.5% quantiles as the lower and upper limits of the 95% credible intervals (CrI), respectively. We declared an effect significant when the corresponding 95% CrI did not include zero. Statistics were detailed for significant interactions only but not for their corresponding primary effects. The mean value and corresponding 95% confidence intervals for the interactions of mixed models were estimated using the “effect” function of the R package “effects” [99]. The values of other explanatory variables were fixed at their means. For further information see [99]. Details on statistical models, including residual analyses that show no violation of model assumptions, are provided in Additional file 4.

## Results

**Fig. 1:**
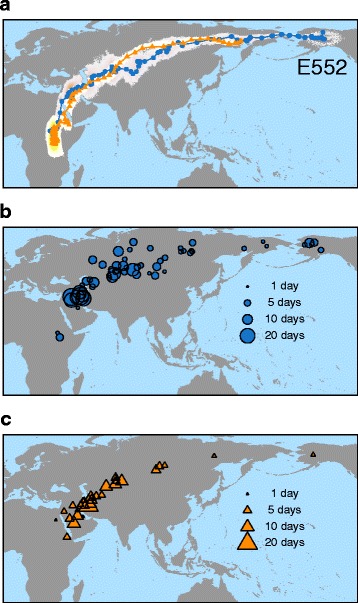
Migration route (**a**) and stopover sites in autumn (**b**) and spring (**c**). **a** Migration route of one northern wheatear from a breeding population in Alaska showing the mean daily location estimates (autumn: *blue*-filled *circles*; spring: *orange*-filled *triangles*) and the 95% CrI of these location estimates (autumn: *pink*; spring: *yellow*). Location estimates for breeding area (65.6° N, 145.4° W; 68.6° N, 149.6° W; U.S.A.) and wintering ground were removed. Furthermore, locations with 24 h daylight at high latitudes in spring are missing due to the dependency of the tracking method on sunrise and sunset times. Additional file 5 provides maps of all successfully tracked individuals. **b**, **c** Mean location estimates of stopovers for all birds tracked during autumn (**b**) and spring (**c**) migration. Individual size of symbols is proportional to duration of stopover

Of the 13 tagged birds that return to their breeding sites in the year after deployment (5 in 2010; 8 in 2014), only eight tracks were obtained because two birds lost their geolocator device, and three individuals could not be recaptured (Additional file 2). Wheatears initiated autumn migration between the 4^th^ and 17^th^ of Aug., crossed the Bering Strait and migrated westwards across Russia and Kazakhstan before heading south and westwards towards Africa in the Middle East (Fig. 1, Table 1, and Additional file 5). After travelling 14,800 ± 1,800 km (*n* = 8), birds arrived at their wintering grounds between the 3^rd^ and 27^th^ of Nov. In spring, birds started their migration between the 24^th^ of Mar. and the 14^th^ of Apr. (Table 1). Geolocation by light failed to estimate locations for birds experiencing 24-h daylight, which was the case for wheatears approaching their breeding area in spring (Fig. 1). Therefore, spring migration routes were not complete and individuals were tracked for 8,000 ± 1,900 km. Most stopover periods occurred along the western part of the route (Fig. 1b, c). The experienced environmental conditions along the route were similar between years, e.g. during autumn migration 2009 and 2013 and during spring 2010 and 2014 (Additional file 6).

**Table 1:** Summary of migration phenology for all tracked Northern wheatears

Bird	Start of autumn migration [Julian day]	Arrival at wintering grounds [Julian day]	Total stopover duration [days]	Total speed of migration in autumn [km/day]	Start of spring migration [Julian day]	Total stopover duration [days]	Total speed of migration in autumn [km/day]
7902	216	318	45	152	98	7	262
7910	233	315	33	163	94	18	279
7916	229	317	38	173	91	25	166
B070	226	315	38	181	112	9	324
E552	216	307	45	136	100	7	271
E553	229	322	53	139	89	18	182
E801	221	315	43	287	90	20	160
E823	227	331	60	247	84	26	198

Note, that tracking towards Alaska in spring ceased after birds entered areas with 24 h daylight. Total stopover duration and total speed of migration were therefore only calculated by departure date from the wintering ground to arrival at the last location estimate during spring migration (cf. Fig. 1 and Additional file 4 for more details)

**Fig. 2:**
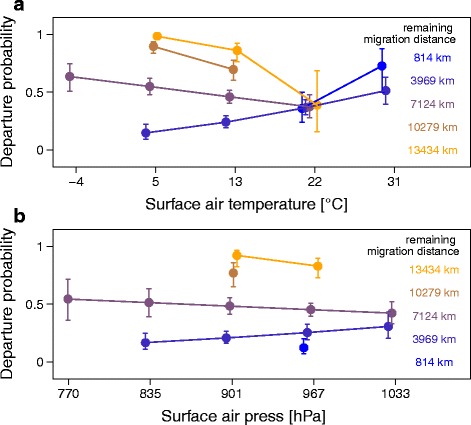
Model predictions on the effect of surface air temperature and of surface air pressure both by remaining migration distance on departure probabilities of 694 autumn migration days. To visualize the effect of both two-way interactions between (**a**) surface air temperature and remaining migration distance as well as (**b**) surface air pressure and remaining migration distance on the departure probability for each day during autumn migration as predicted by the model (Table 2), explanatory variables were each summarized into five categories. Within each temperature category, values were slightly shifted along the x-axis to avoid overlapping. Given are the mean effects with the 95% CI. The y-axis shows the departure probability ranging from 0 to 1. Different colours indicate different remaining migration categories. Model predictions are only shown for surface air temperatures and surface air pressure actually experienced by the wheatears in the wild

**Fig. 3:**
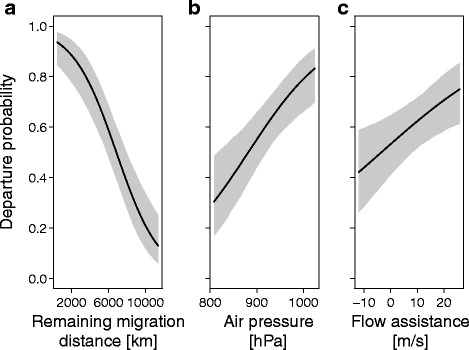
Model predictions on the effect of (**a**) remaining migration distance, (**b**) air pressure, and (**c**) flow assistance on departure probabilities of 297 spring migration days. Given are the fitted values (*black line*), with the 95% CrI in *grey*. To show the effect of the remaining migration distance, the precipitation was set to 0.6 kg/m^2^/s, the air pressure to 943 hPa, the flow assistance to 4.0 m/s, and the wind speed to 4.6 m/s, which were the mean values as experienced by the eight birds in spring. To show the effect of air pressure and flow assistance on bird departure probability, the remaining migration distance was set to 6,880 km, with corresponding values as given above

In autumn, departure probability generally decreased with increasing surface air temperature, improving flow assistance, and decreasing remaining migration distance (Table 2). We found no significant effect of the main effects precipitation, surface air pressure, and surface wind speed on the individual departure probability (Table 2). The interaction between the surface air temperature and the remaining migration distance on the birds departure probability was found significant, indicating that the reaction to temperature changed with the remaining migration distance (Table 2; Fig. 2). During their first 8,000 km wheatears commonly experienced temperatures ranging from -5 °C to +20 °C (Additional file 7). Surface air temperature significantly affected departure probability with low temperatures being associated with high departure probabilities during the first two thirds of migration distance (Fig. 2). In contrast, relatively low temperatures were associated with low departure probabilities and high temperatures with high probabilities along the remaining one third of the migration distance (Fig. 2). Note that wheatears did not encounter temperatures above 20 °C on their first two thirds of migration and below 5 °C on their final third of migration. Corresponding model predictions should therefore be treated with caution. The interaction between the surface air pressure and the remaining migration distance was significant, suggesting that low surface air pressures were generally associated with high departure probabilities at the beginning of autumn migration, while far from Alaska departure probability increased with increasing air pressures (Table 2, Fig. 2). In spring, departure probability increased with rising surface air pressure, improving flow assistance, and decreasing remaining migration distance (Table 2, Fig. 3). No significant effects were found for precipitation, surface wind speed, or any other interactions (Table 2).

**Table 2:** Daily departure probabilities

	Autumn		Spring	
Parameter	Estimate	95% CrI	Estimate	95% CrI
Intercept	-0.25	-0.46 – 0.04	0.39	-0.05 – 0.82
Surface air temperature	**-0.30**	**-0.56 – -0.04**	NA	NA
Precipitation	0.14	-0.05 – 0.32	0.02	-0.47 – 0.46
Surface air pressure	-0.09	-0.32 – 0.13	**0.70**	**0.34 – 1.07**
Surface wind speed	0.00	-0.17 – 0.19	-0.30	-0.61 – 0.02
Flow assistance	**-0.33**	**-0.53 – -0.12**	**0.42**	**0.14 – 0.72**
Remaining migration distance	**0.98**	**0.73 – 1.23**	**-1.34**	**-1.81 – -0.86**
Surface air temperature-*-remaining migration distance	**-0.83**	**-1.11 – -0.57**	NA	NA
Precipitation-*-remaining migration distance	0.07	-0.13 – 0.25	0.06	-0.48 – 0.57
Surface air pressure-*- remaining migration distance	**-0.30**	**-0.57 – -0.01**	0.03	-0.50 – 0.60
Surface wind speed -*- remaining migration distance	0.20	-0.01 – 0.41	0.04	-0.52 – 0.57
Flow assistance-*- remaining migration distance	0.06	-0.20 – 0.31	-0.23	-0.70 – 0.24
Standard deviation of random factor year (2009 and 2013)	<0.0001		0.21	
Sample size (number of days on migration of all birds)	694		297	
Marginal R^2^	0.33		0.46	
Conditional R^2^	0.33		0.47	

Results of the model on the individual daily departure probability along the migration route for autumn and spring. Environmental variables describe conditions at sunset. In spring, air temperature was collinear with remaining migration distance and therefore air temperature was removed from the model. Estimates and 95% CrI are given for each environmental variable. 95% CrI not including zero are given in bold. More details, including scale parameter to assess overdispersion, goodness of fit, etc., are given in Additional file 4

**Fig. 4:**
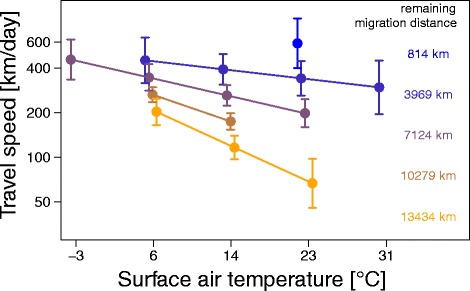
Model predictions on the effect of surface air temperature by remaining migration distance on travel speed during 303 travel days in autumn. To visualize the effect of the two-way interaction between surface air temperature and remaining migration distance on the travel speed for each travel day during autumn migration as predicted by the model (Table 3), explanatory variables were each summarized into five categories. Within each temperature category, values were slightly shifted along the x-axis to avoid overlapping. Given are the mean effects with the 95% CI. The y-axis shows the estimated mean travel speed for the different categories. Different colours indicate different remaining migration categories. Model predictions are only shown for surface air temperatures actually experienced by the wheatears in the wild

**Fig. 5:**
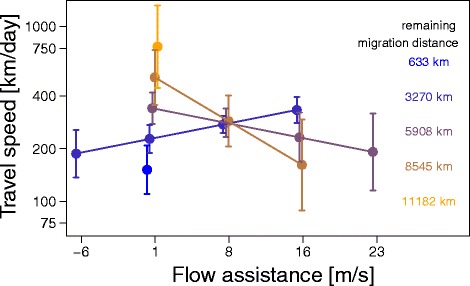
Model predictions on the effect of flow assistance by remaining migration distance on travel speed during 153 travel days in spring. To visualize the effect of the two-way interaction between flow assistance and remaining migration distance on the travel speed for each travel day during spring migration as predicted by the model (Table 3), explanatory variables were each summarized into five categories. Within each flow assistance category, values were slightly shifted along the x-axis to avoid overlapping. Given are the mean effects with the 95% CI. The y-axis shows the estimated mean travel speed for the different categories. Different colours indicate different remaining migration categories. Consider that the migration distance tracked during spring differed between individuals, because birds’ locations were not determinable when they were exposed to 24 h of daylight, cf. Fig. 1 and Additional file 5. Last location estimates were still 650 to 6,000 km, with a mean value of 3,400 km, away from the breeding area. Model predictions are only shown for flow assistance actually experienced by the wheatears in the wild

In autumn, travel speed increased with air pressure and was generally higher, if surface air temperature was low (Table 3). The effect of the surface air temperature on travel speed diminished, once the birds approached the wintering grounds (Fig. 4). In spring, precipitation had an apparent positive effect on the travel speed at the beginning of spring migration, whereas the reverse effect was observed during the remaining half of spring migration. However, occurrence of precipitation was strongly biased towards the second half of spring migration, which may have potentially influenced the interaction (Additional file 8). The significant interaction between the flow assistance and the remaining migration distance indicated that birds took advantage of flow assistance as they approached the breeding areas (Fig. 5). Additional file 8 shows the flow assistance experienced by wheatears.

**Table 3:** Variation in daily travel speed

	Autumn		Spring	
Parameter	Estimate	95% CrI	Estimate	95% CrI
Intercept	**2.45**	**2.37 – 2.52**	**2.69**	**2.54 – 2.83**
Air temperature	**-0.12**	**-0.18 – -0.07**	NA	NA
Precipitation	-0.01	-0.06 – 0.05	**0.36**	**0.12 – 0.59**
Air pressure	**0.08**	**0.03 – 0.12**	-0.01	-0.11 – 0.09
Flow assistance	-0.04	-0.12 – 0.04	**-0.13**	**-0.23 – -0.04**
Remaining migration distance	**-0.16**	**-0.22 – -0.10**	**0.27**	**0.16 – 0.37**
Air temperature-*-remaining migration distance	**-0.06**	**-0.11 – -0.01**	NA	NA
Precipitation-*-remaining migration distance	0	-0.05 – 0.05	**0.35**	**0.19 – 0.50**
Air pressure-*- remaining migration distance	0.03	-0.01– 0.07	-0.06	-0.14 – 0.02
Flow assistance-*- remaining migration distance	-0.03	-0.10 – 0.03	**-0.14**	**-0.22 – -0.07**
Standard deviation of random factor year (2009 and 2013)	<0.0001		<0.0001	
Standard deviation of residuals	0.30		0.36	
Sample size (number of stopover days of all birds)	303		153	
Marginal R^2^	0.22		0.22	
Conditional R^2^	0.22		0.22	

Results of the model on the individual daily travel speed along the migration route for autumn and spring. Environmental variables describe conditions at sunset. In spring, air temperature was collinear with remaining migration distance and therefore air temperature was removed from the model. Estimates and 95% CrI are given for each environmental variable. 95% CrI not including zero are given in bold. More details, including scale parameter to assess overdispersion, goodness of fit, etc., are given in Additional file 4

**Fig. 6:**
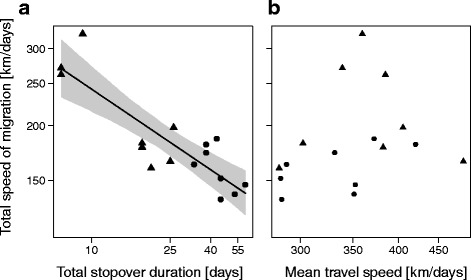
Relationship between total speed of migration and (**a**) total stopover duration, (**b**) mean travel speed. In (**a**), the *black line* is used to visualize the significant relationship between the total speed of migration and total stopover duration, with the corresponding 95% CrI in *grey*. **b** The total speed of migration was not significantly affected by the mean travel speed. All axes are on a log_10_-scale. Spring (*triangles*) and autumn (*dots*) data were pooled (see Results). Notably, the tracking towards Alaska in spring ceased when birds experienced 24 h of daylight. Total speed of migration and mean travel speed were therefore only calculated by departure date from the wintering ground to arrival date at the last location estimate during spring migration, cf. Fig. 1. Here we assume that the overall mechanism shaping total speed of migration is unlikely to change in its nature along the migration route. If so, pooling the autumn and the incomplete spring data should not influence how total stopover duration affects total speed of migration

Neither the timing of autumn departure nor the total migration distance were related to arrival timing at the wintering grounds (Wilcoxon signed rank sum test: S > 61, *p* > 0.5, *n* = 8). As wheatears experienced 24 h of daylight at locations close to the breeding areas in spring, arrival timing could not be related either to start of migration or to total migration distance. The total stopover duration had a significant negative effect on the total speed of migration (LMM: log_10_(total stopover duration), 95% CrI: -0.4 – -0.20 d, *n* = 16; in a more complex model, the additional fixed effect ‘season’ was not significant, 95% CrI: -0.17–0.01, and therefore omitted in the final model; Table 4, Fig. 6a, Additional file 4). Due to the truncated tracks in spring, we would not compare the total stopover duration between seasons (Fig. 1, Additional file 5). In autumn, individuals showed a median stopover duration of 2.7 days per 1,000 km (25% quantile: 2.5 days, 75% quantile: 3.7 days), compared to 2.3 days (1.2 days, 2.8 days) in spring (Wilcoxon signed-rank test comparing autumn with spring values: V = 28, *p* = 0.20, *n* = 8). We found no effect of mean travel speed on total speed of migration (LMM: log_10_(mean travel speed), 95% CrI: -0.50–0.99 km/day, *n* = 16; Table 4, Fig. 6b). However, in this model the effect of the season was significant (95% CrI: 0.02–0.22) with higher values during spring migration (Table 4).

**Table 4:** Variation in total speed of migration

	Total stopover duration [days]		Mean travel speed [km/days]	
Parameter	Estimate	95% CrI	Estimate	95% CrI
Intercept	**2.69**	**2.54 – 2.84**	1.68	-0.35 – 3.47
Log10 (predictor)	**-0.31**	**-0.41 – -0.20**	0.20	-0.50 – 0.99
Season			**0.12**	**0.02 – 0.22**
Standard deviation of random factor individual bird	0.004		<0.001	
Standard deviation of residuals	0.055		0.09	
Sample size (number of migrations)	16		16	
Marginal R^2^	0.74		0.34	
Conditional R^2^	0.74		0.34	

Results of two linear mixed effect models explaining between-individual variation in total speed of migration by the predictor ‘total stopover duration’ and by the predictors ‘mean travel speed’ and ‘season’. In the first model, the fixed effect ‘season’ was not significant, 95% CrI: -0.17–0.01 and therefore omitted in the final model. Estimates and 95% CrI are given for each environmental variable. 95% CrI not including zero are given in bold

## Discussion

In this study, wheatears were tracked with light-level geolocators from their breeding areas in Alaska to their wintering grounds in eastern Africa and back (Fig. 1, Additional file 5). Despite the relatively low spatial resolution of both the tracking [70, 71, 100] and the environmental data [84], as well as the limited sample size of eight individuals, our results show clear dependencies of migratory behaviour and perceived environmental conditions. Most importantly, we found that individual birds adjusted their departure decisions and travel speeds to the encountered environmental conditions along their 15,000 km migration routes (Figs. 2, 3, 4 and 5). These adjustments were flexible in space and time indicating that the individual phenotypic reactions to the same set of environmental conditions may change along the migration route. Hence, we cannot assume that reactions to changes in the environment at specific sites somewhere along the pathway are general behavioural patterns that can be extrapolated to the entire migration route. In addition, we showed that variation in total stopover duration is the main factor in determining total speed of migration in the tracked wheatears. Hence, total stopover duration crucially affected the individual’s phenology (Fig. 6). Detailed understanding on how migratory birds react to changes in the environment along their entire migration route and how this affects total speed of migration is essential to improve our ability to predict the consequences of continuing and rapid environmental changes on bird migration.

Wheatears in this study responded differently to prevailing environmental conditions in autumn and spring (Figs. 2, 3 and 4). In autumn, birds did not time their migratory flights in accordance with increasing flow assistance, which would maximize their travel speed [65]. No association was found with wind speed and precipitation (Table 2), contrasting findings of former studies [58, 59, 64, 65, 101, 102]. One reason could be, that the weather conditions at a local scale were different to the modelled regional weather conditions used in this study, or that other extrinsic conditions, e.g. surface air temperature, or intrinsic factors, e.g. body condition, had a stronger effect on birds’ decision to resume migration than precipitation and/or wind [37, 103]. The latter has often been observed in cases where flow assistance was not significantly adverse [54, 58, 104–106]. In our study flow assistance was indeed mostly favourable (> -5 m/s; Additional file 9). At the beginning of autumn migration, the tracked wheatears resumed migration and travelled long distances during conditions characterised by low surface air temperatures and low surface air pressure (Figs. 2 and 4). We know that energy costs associated with thermoregulation during stopover are significantly minimized by avoiding cold temperature on the ground [57], flying long stretches towards likely warmer regions and possibly eluding fronts potentially carrying rain. During stopover diurnal movements, e.g. in search for food, were shown to be inversely associated with rain [36]. Due to increased mortality and energy expenditure, is expected to be costly to migrating during rain events [59]. Hence, wheatears seem to minimize energy expenditure at stopovers during autumn. Models on departure probability and travel speed include predictions for environmental conditions not experienced by wheatears en route, e.g. high temperatures at the beginning and low temperatures at the final part of autumn migration (Figs. 2 and 4). This change in the distribution of environmental predictors (e.g. air temperature) with remaining migration distance may affect the significant interactions. Nevertheless, neglecting these extreme conditions still showed that wheatears adjusted their departure decision and travel speed to a similar set of environmental conditions depending on the remaining migration distance (Figs. 2 and 4).

In spring, surface air pressure positively influenced departure probability (Table 2). This affect is most likely attributed to the fact that high surface air pressures generally represents favourable conditions for migration [107]. Flow assistance influenced the departure probability in spring (Fig. 3), supporting the findings of multiple other studies [58, 59, 64, 65, 101, 102]. Both, minimization of energy or time can lead to such behavioural pattern [41]. Interestingly, reducing stopovers (Fig. 1) and increasing departure probabilities along the migration route (Fig. 3) weakened the importance of stopover duration on the total speed of migration but simultaneously strengthened the significance of travel speed. Therefore, the increase in exploiting flow assistance with season (Fig. 5) was likely a behavioural adjustment to speed up migration, achieved by an increase in the distance travelled per night. Why flow assistance had the opposite effect on travel speed at the beginning of spring (Fig. 5) remains unknown. In addition to weather conditions, migrants are able to use cues and experience associated to the landscape of stopover sites to adjust migration phenology [108, 109]. The facts that we found a concentration of stopover sites just before the Arabian and Sahara deserts in autumn as well as in spring (Fig. 1) indicate that these sites may have been used to prepare and/or recover from crossings those ecological barriers. It remains to be investigated whether the few stopovers in the far-east of Russia during autumn migration indicate infrequent patches of suitable habitats for wheatears to refuel, as demonstrated for bluethroats (*Lusinia sveccia*) [110], or highly favourable feeding conditions allowing quick replenishment of fuel resources used during the previous migratory flight.

The correct timing of arrival at breeding areas is predicted to have generally stronger, more immediate fitness consequences than the timing of arrival at the wintering grounds [67]. Hence, minimizing time rather than energy use is likely more beneficial in spring than during autumn migration [35, 43]. This trade-off between minimizing either time or energy and the resulting seasonally different selection pressures should lead to seasonally different reactions to the environment, as observed in the wheatears of this study (Figs. 2, 3 and 4).

Total stopover duration was a significant factor explaining total speed of migration (Fig. 6), which is in line with a recent tracking study of greater white-fronted geese (*Anser albifrons*) [68]. In contrast to other migration studies in songbirds, in which the seasonal start of migration was correlated with the arrival date at the final destination [20, 25–29, 78], total stopover duration in the tracked wheatears was found more important in determining arrival dates. It seems possibly that the extremely long distance of the migration route and the resulting high number of stopovers (Fig. 1) moderates the effect of the onset of migration on arrival timing (Table 1) in comparison to shorter distance migrants that rest at fewer stopover sites [29, 111, 112]. The arrival timing at migratory destination, i.e. breeding area or wintering ground, is also explained by the environmental conditions experienced somewhere en route [10, 113]. Thus, environmental conditions affecting departure probability and travel speed (Figs. 2, 4 and 5) might have a stronger cumulative effect on the phenology in wheatears breeding in Alaska than in shorter distance migrants. In the former, the cumulative effect of the environment on the total stopover duration is, therefore, likely an important driver of the variation in arrival timing via total speed of migration, cf. Fig. 6.

In addition to the variation in the environment affecting departure probability and travel speed (Tables 2 and 3), the endogenously controlled changes in bird physiology throughout an annual cycle influences the overall rate of energy accumulation [114–116]. However, the within-season variation in this rate cannot be attributed to an endogenously controlled change in the motivation to refuel over the season [117]; therefore, variation in the environment is the most likely source for the variation in the rate of accumulating energy within a season. The innate migration programme also contributes in the regulation of departure probability, which at least in spring increases with the progression of the season [117] and with the birds getting closer to their breeding area (Fig. 3). Further support for the importance of the innate migration program on birds’ phenology comes from a high within-individual repeatability in the timing of onset of migration and arrival at the migratory destination across years [27, 30]. Therefore, evolutionary processes acting on onset of spring migration, total speed of migration, and/or total migration distance are likely required for birds to adapt to future environmental conditions to arrive on time [5, 6, 22, 118]. However, an adaptive evolutionary response to an environmental change is not immediate [6, 119], and the extent of delay is a function of the rate of contemporary microevolution and generation times, i.e., at least one year in birds [120]. Because climate change is characterized by an increase in temperature combined with greater between-year variability [121], among other variables, microevolutionary responses are unlikely to explain alone the twice a year seasonal variation that occurs in bird phenology. Therefore, phenotypic flexibility is generally accepted as an important proximate cause underlying recent changes in the timing of migration [20, 118, 122].

To ultimately assess the predictions, that phenotypic flexibility is one of the major drivers for the current shifts in migratory phenology [123], we need to find strong spatial autocorrelations along the migratory routes allowing migrants en-route to predict future conditions on the breeding area and/or wintering ground [33]. Furthermore, we need a deeper understanding on how birds respond to these changes on site. Our results suggests that wheatears potentially adjusted total speed of migration to changes in the environment via a plastic phenotypic response in the departure probability, affecting stopover duration, and the travel speed. However, endogenous constraints and a spatial-specific reaction norm (e.g. not taking advantage of tail wind conditions during the first half of spring migration; Fig. 5) may limit the magnitude of this reaction (e.g. departure probability; Table 2, Fig. 2). Thus, changes in a certain environmental parameter may potentially evoke a strong response at one site, but a weaker or even a different reaction at another site. To improve understanding how phenological change [123] is proximately caused [15, 20], an elaborate effort to study migrants’ reaction to environmental conditions along the entire migration route as well as at the breeding areas and wintering grounds is indispensable.

## Conclusion

Our results are based on location estimates derived from light-level geolocation data recorded during autumn and spring migration of eight wheatears breeding in Alaska. The presented results needs to be treated cautiously given the inherent problems of accuracy and precision in light-level geolocation as well as the coarse resolution of the environmental data [84]. However, and despite these issues we found that wheatears phenotypically adjusted their departure probability and travel speed according to environmental conditions experienced along the entire migration route. More specifically, individuals showed flexible adjustments over time and space and between the seasons. The consideration of this complex phenotypic flexibility in bird reaction norms to the environment and the importance of total stopover duration on total speed of migration are essential to understand the influence of climate change on migration in general and timing in particular. Such knowledge might be crucial to understand why there is an apparent phenological mismatch in some [13–17] but not all species [18, 19] and their food resources, e.g. if the importance of total stopover duration on total speed of migration varies between species. In some species, total speed of migration might be unrelated or only weakly associated to variation in total stopover duration [29]. In this case, and because environmental variation experienced during migration is unlikely to alter phenology via a change in total speed of migration, changes at the wintering grounds have likely the highest significant impact on their arrival times at the breeding area. These changes concern location of wintering grounds and onset of spring migration. In other species, total speed of migration is a function of total stopover duration (Fig. 6). In those, environmental conditions can have a significant effect on departure probability from stopover sites, which in turn shapes arrival timing at the migratory destination via total stopover duration. There are two non-mutually exclusive, potential solutions of how birds could overcome the current phenological mismatch with the primary food source at the breeding area. Either the phenotypic responses to ongoing changes in the environment are sufficient or evolutionary processes are required for migration phenology to keep pace. To predict the natural solutions to this challenge, future models need to incorporate the spatiotemporal-specific reaction norm with the environment and the species-specific significance of total stopover duration on total speed of migration.

## Additional files

Additional file 1: Individual data of birds, table. (DOCX 39 kb)
Additional file 2: Recapture probability of tagged Northern Wheatears, documentation. (DOCX 159 kb)
Additional file 3: R code for analyzing light-level geolocation data, documentation. (DOCX 186 kb)
Additional file 4: R code for analyzing phenotypic response to environmental cues, documentation. (DOCX 147 kb)
Additional file 5: Migration routes of the Northern Wheatears, figures. (DOCX 239 kb)
Additional file 6: Environmental conditions during autumn and spring migration, figures. (DOCX 108 kb)
Additional file 7: Air temperature over remaining migration distance in autumn, figure. (DOCX 67 kb)
Additional file 8: Precipitation and flow assistance before performing a migratory flight in spring, figures. (DOCX 63 kb)
Additional file 9: Flow assistance during autumn migration, figure. (DOCX 47 kb)

## References

[1] Karl TR, Trenberth KE (2003). Modern global climate change. Science.

[2] Thackeray SJ, Henrys PA, Hemming D, Bell JR, Botham MS, Burthe S, Helaouet P, Johns DG, Jones ID, Leech DI (2016). Phenological sensitivity to climate across taxa and trophic levels. Nature.

[3] Walther G-R, Post E, Menzel A, Parmesan C, Beebee TJC, Fromentin J-M, Hoegh-Guldberg O, Bairlein F (2002). Ecological responses to recent climate change. Nature.

[4] Jenni L, Kéry M (2003). Timing of autumn bird migration under climate change: advances in long-distance migrants, delays in short-distance migrants. Proc R Soc Lond B.

[5] Gill JA, Alves JA, Sutherland WJ, Appelton GF, Potts PM, Gunnarsson TG (2014). Why is timing of bird migration advancing when individuals are not?. Proc R Soc Lond B.

[6] Visser ME, Gienapp P, Husby A, Morrisey M, de la Hera I, Pulido F, Both C (2015). Effects of spring temperatures on the strength of selection on timing of reproduction in a long-distance migratory bird. PLoS Biol.

[7] Edwards M, Richardson AJ (2004). Impact of climate change on marine pelagic phenology and trophic mismatch. Nature.

[8] Both C, van Asch M, Bijlsma RG, van den Burg AB, Visser ME (2009). Climate change and unequal phenological changes across four trophic levels: constraints or adaptations?. J Anim Ecol.

[9] van Gils JA, Lisovski S, Lok T, Meissner W, Ożarowska A, de Fouw J, Rakhimberdiev E, Soloviev MY, Piersma T, Klaassen M (2016). Body shrinkage due to Arctic warming reduces red knot fitness in tropical wintering range. Science.

[10] Hüppop O, Hüppop K (2003). North Atlantic Oscillation and timing of spring migration in birds. Proc R Soc Lond B.

[11] Jonzén N, Lindén A, Ergon T, Knudsen E, Vik JO, Rubolini D, Piacentini D, Brinch C, Spina F, Karlsson L (2006). Rapid advance of spring arrival dates in long-distance migratory birds. Science.

[12] Bairlein F (2016). Migratory birds under threat. Science.

[13] Møller AP, Rubolini D, Lehikoinen E (2008). Populations of migratory bird species that did not show a phenological response to climate change are declining. PNAS.

[14] Both C, Van Turnhout CAM, Bijlsma RG, Siepel H, Van Strien AJ, Foppen RPB (2010). Avian population consequences of climate change are most severe for long-distance migrants in seasonal habitats. Proc R Soc Lond B.

[15] Vickery JA, Ewing SR, Smith KW, Pain DJ, Bairlein F, Škorpiloá J, Gregory RD (2014). The decline of Afro-Palaearctic migrants and an assessment of potential causes. Ibis.

[16] Sanderson FJ, Donald PF, Pain DJ, Burfield IJ, van Bommel FPJ (2006). Long-term population declines in Afro-Palearctic migrant birds. Biol Cons.

[17] Rushing CS, Ryder TB, Marra PP. Quantifying drivers of population dynamics for a migratory bird throughout the annual cycle. Proc R Soc Lond B. 2016; 283.10.1098/rspb.2015.2846. 10.1098/rspb.2015.2846 PMC4795038 26817774

[18] Dunn PO, Møller AP (2014). Changes in breeding phenology and population size of birds. J Anim Ecol.

[19] Senner NR, Stager M, Sandercock BK (2017). Ecological mismatches are moderated by local conditions for two populations of a long-distance migratory bird. Oikos.

[20] Knudsen E, Lindén A, Both C, Jonzén N, Pulido F, Saino N, Sutherland WJ, Bach LA, Coppack T, Ergon T (2011). Challenging claims in the study of migratory birds and climate change. Biol Rev.

[21] Coppack T, Both C (2002). Predicting life-cycle adaptations of migratory birds to global climate change. Ardea.

[22] Durant JM, Hjermann DO, Otterseon G, Stenseth NC (2007). Climate and the match or mismatch between predator requirements and resource availability. Clim Res.

[23] Studds CE, Marra PP (2011). Rainfall-induced changes in food availability modify the spring departure programme of a migratory bird. Proc R Soc Lond B.

[24] Cooper NW, Sherry TW, Marra PP (2015). Experimental reduction of winter food decreases body condition and delays migration in a long-distance migratory bird. Ecol.

[25] Schmaljohann H, Meier C, Arlt D, Bairlein F, van Oosten HH, Morbey YE, Åkesson S, Buchmann M, Chernetsov N, Desaever R (2016). Proximate causes of avian protandry differ between subspecies with contrasting migration challenges. Behav Ecol.

[26] Tøttrup AP, Klaassen RHG, Strandberg R, Thorup K, Willemoes Kristensen M, Søgaard Jørgensen P, Fox JW, Afanasyev V, Rahbek C, Alerstam T (2012). The annual cycle of a trans-equatorial Eurasian-African passerine migrant: different spatio-temporal strategies for autumn and spring migration. Proc R Soc Lond B.

[27] Stanley CQ, MacPherson M, Fraser KC, McKinnon EA, Stutchbury BJM (2012). Repeat tracking of individual songbirds reveals consistent migration timing but flexibility in route. PLoSONE.

[28] Jahn AE, Cuteo VR, Fox JW, Husak MS, Kim DH, Landoll DV, Ledezma JP, LePage HK, Levey DJ, Murphy MT, Renfrew RB (2013). Migration timing and wintering areas of three species of flycatchers (*Tyrannus*) breeding in the Great Plains of North America. Auk.

[29] Ouwehand J, Both C (2017). African departure rather than migration speed determines variation in spring arrival in pied flycatchers. J Anim Ecol.

[30] Conklin JR, Battley PF, Potter MA (2013). Absolute consistency: Individual versus population variation in annual-cycle schedules of a long-distance migrant bird. PLoSONE.

[31] McKinnon EA, Stanley CQ, Stutchbury BJM (2015). Carry-over effects of nonbreeding habitat on start-to-finish spring migration performance of a songbird. PLoSONE.

[32] Senner NR, Hochachka WM, Fox JW, Afanasyev V (2014). An exception to the rule: carry-over effects do not accumulate in a long-distance migratory bird. PLoSONE.

[33] Both C (2010). Flexibility of timing of avian migration to climate change masked by environmental constraints en route. Curr Biol.

[34] Marra PP, Francis CM, Mulvihill RS, Moore FR (2005). The influence of climate on the timing and rate of spring bird migration. Oecol.

[35] Alerstam T, Lindström Å, Gwinner E (1990). Optimal bird migration: the relative importance of time, energy, and safety. Bird migration: physiology and ecophysiology.

[36] Smith AD, McWilliams SR (2014). What to do when stopping over: behavioral decisions of a migrating songbird during stopover are dictated by initial change in their body condition and mediated by key environmental conditions. Behav Ecol.

[37] Jenni L, Schaub M, Berthold P, Gwinner E, Sonnenschein E (2003). Behavioural and physiological reactions to environmental variation in bird migration: a review. Avian migration.

[38] Deppe JL, Ward MP, Bolus RT, Diehl RH, Celis-Murillo A, Zenzal TJ, Moore FR, Benson TJ, Smolinsky JA, Schofield LN (2015). Fat, weather, and date affect migratory songbirds’ departure decisions, routes, and time it takes to cross the Gulf of Mexico. PNAS.

[39] Crysler ZJ, Ronconi RA, Taylor PD (2016). Differential fall migratory routes of adult and juvenile Ipswich Sparrows (*Passerculus sandwichensis princeps*). Mov Ecol.

[40] Mitchell GW, Woodworth BK, Taylor PD, Norris DR (2015). Automated telemetry reveals age specific differences in flight duration and speed are driven by wind conditions in a migratory songbird. Mov Ecol.

[41] Liechti F, Bruderer B (1998). The relevance of wind for optimal migration theory. J Avian Biol.

[42] Alerstam T (2001). Detours in bird migration. J Theor Biol.

[43] Karlsson H, Nilsson C, Bäckman J, Alerstam T (2012). Nocturnal passerine migrants fly faster in spring than in autumn: a test of the time minimization hypothesis. Anim Behav.

[44] Sergio F, Tanferna A, De Stephanis R, Jiménez LJ, Blas J, Tavecchia G, Preatoni D, Hiraldo F (2014). Individual improvements and selective mortality shape lifelong migratory performance. Nature.

[45] Chevallier D, Handrich Y, Georges J-Y, Baillon F, Brossault P, Aurouet A, Le Maho Y, Massemin S (2010). Influence of weather conditions on the flight of migrating black storks. Proc R Soc Lond B.

[46] Klaassen RHG, Hake M, Strandberg R, Alerstam T (2010). Geographical and temporal flexibility in the response to crosswinds by migrating raptors. Proc R Soc Lond B.

[47] Flack A, Fiedler W, Blas J, Pokrovsky I, Kaatz M, Mitropolsky M, Aghababyan K, Fakriadis I, Makrigianni E, Jerzak L (2016). Costs of migratory decisions: a comparison across eight white stork populations. Sci Adv.

[48] Mandel JT, Bildstein KL, Bohrer G, Winkler DW (2008). Movement ecology of migration in turkey vultures. PNAS.

[49] Vansteelant WMG, Bouten W, Klaassen RHG, Koks BJ, Schlaich AE, van Diermen J, Van Loon EE, Shamoun-Baranes J (2015). Regional and seasonal fl ight speeds of soaring migrants and the role of weather conditions at hourly and daily scales. J Avian Biol.

[50] Vansteelant WMG, Shamoun-Baranes J, van Manen W, van Diermen J, Bouten W (2016). Seasonal detours by soaring migrants shaped by wind regimes along the East Atlantic flyway. J Anim Ecol.

[51] Åkesson S, Bianco G, Hedenström A (2016). Negotiating an ecological barrier: crossing the Sahara in relation to winds by common swifts. Philos T Roy Soc B.

[52] Wikelski M, Kays R, Kasdin NJ, Thorup K, Smith JA, Swenson GW (2007). Going wild: what a global small-animal tracking system could do for experimental biologists. J Exp Biol.

[53] McKinnon EA, Fraser KC, Stutchbury BJM (2013). New discoveries in landbird migration using geolocators, and a flight plan for the future. Auk.

[54] Schmaljohann H, Korner-Nievergelt F, Naef-Daenzer B, Nagel R, Maggini I, Bulte M, Bairlein F (2013). Stopover optimization in a long-distance migrant: the role of fuel load and nocturnal take-off time in Alaskan northern wheatears (Oenanthe oenanthe). Front Zool.

[55] Bairlein F, Norris DR, Nagel R, Bulte M, Voigt CC, Fox JW, Hussell DJT, Schmaljohann H (2012). Cross-hemisphere migration of a 25-gram songbird. Biol Lett.

[56] Schmaljohann H, Fox JW, Bairlein F (2012). Phenotypic response to environmental cues, orientation and migration costs in songbirds flying halfway around the world. Anim Behav.

[57] Wikelski M, Tarlow EM, Raim A, Diehl RH, Larkin RP, Visser GH (2003). Costs of migration in free-flying songbirds. Nature.

[58] Erni B, Liechti F, Underhill LG, Bruderer B (2002). Wind and rain govern the intensity of nocturnal bird migration in central Europe - a log-linear regression analysis. Ardea.

[59] Schaub M, Liechti F, Jenni L. Departure of migrating European robins, *Erithacus rubecula*, from a stopover site in relation to wind and rain. Anim Behav. 2004;229–237.

[60] Dau CP (1992). The fall migration of pacific flyway Brent Branta bernicla in relation to climatic conditions. Wildfowl.

[61] Richardson WJ, Gwinner E (1990). Timing of bird migration in relation to weather: updated review. Bird migration.

[62] Zehnder S, Åkesson S, Liechti F, Bruderer B (2001). Nocturnal autumn bird migration at Falsterbo, south Sweden. J Avian Biol.

[63] Delingat J, Bairlein F, Hedenström A (2008). Obligatory barrier crossing and adaptive fuel management in migratory birds: the case of the Atlantic crossing in Northern Wheatears (*Oenanthe oenanthe*). Behav Ecol Sociobiol.

[64] Åkesson S, Hedenström A (2000). Wind selectivity of migratory flight departures in birds. Behav Ecol Sociobiol.

[65] Liechti F (2006). Birds: blowin’ by the wind?. J Ornithol.

[66] Müller F, Taylor PD, Sjöberg S, Muheim R, Tsvey A, Schmaljohann H (2016). Towards a conceptual framework for explaining variation in the nocturnal departure time of songbird migrants. Mov Ecol.

[67] Kokko H (1999). Competition for early arrival in birds. J Anim Ecol.

[68] Kölzsch A, Müskens GJDM, Kruckenberg H, Glazov P, Weinzierl R, Nolet BA, Wikelski M (2016). Towards a new understanding of migration timing: slower spring than autumn migration in geese reflects different decision rules for stopover use and departure. Oikos.

[69] Wilson RP, Ducamp JJ, Rees G, Culik BM, Niekamp K, Priede IM, Swift SM (1992). Estimation of location: global coverage using light intensity. Wildlife telemetry: remote monitoring and tracking of animals.

[70] Hill RD, Boeuf L, Burney J, Laws RM (1994). Theory of geolocation by light levels. Elephant seals: population ecology, behaviour, and physiology.

[71] Lisovski S, Hewson CM, Klaassen RHG, Korner-Nievergelt F, Kristensen MW, Hahn S (2012). Geolocation by light: accuracy and precision affected by environmental factors. Meth Ecol Evol.

[72] Rakhimberdiev E, Senner NR, Verhoeven MA, Winkler DW, Bouten W, Piersma T (2016). Comparing inferences of solar geolocation data against high-precision GPS data: annual movements of a double-tagged black-tailed godwit. J Avian Biol.

[73] Sumner MD, Wotherspoon SJ, Hindell MA (2009). Bayesian estimation of animal movement from archival and satellite tags. PLoSONE.

[74] Lisovski S, Gosbell K, Christie M, Hoye BJ, Klaassen M, Steward ID, Taysom AJ, Minton C (2016). Movement patterns of Sanderling (*Calidris alba*) along the East Asian Australasian flyway and a comparison of methods to identify crucial areas for conservation. Emu.

[75] R Core Team (2015). R: a language and environment for statistical computing.

[76] Wotherspoon SJ, Sumner MD, Lisovski S. R package SGAT: Solar/Satellite geolocation for animal tracking. GitHub Repository - http://github.com/swotherspoon/sgat 2013. Accessed 29 May 2015.

[77] Wotherspoon SJ, Sumner M, Lisovski S (2013). BAStag: basic data processing for light based geolocation archival tags.

[78] Yamaura Y, Schmaljohann H, Lisovski S, Senzaki M, Kawamura K, Fujimaki Y, Nakamura F (2016). Tracking the Stejneger’s stonechat *Saxicola stejnegeri* along the East Asian–Australian flyway from Japan via China to Southeast Asia. J Avian Biol.

[79] Salewski V, Flade M, Poluda A, Kiljan G, Liechti F, Lisovski S, Hahn S (2013). An unknown migration route of the ‘globally threatened’ aquatic warbler revealed by geolocators. J Ornithol.

[80] Cramp S (1988). Handbook of the birds of Europe, the Middle East and North Africa. The birds of the Western Palearctic.

[81] Green M, Alerstam T, Clausen P, Drent R, Ebbinge BS (2002). Dark-bellied Brent Geese Branta bernicla bernicla, as recorded by satellite telemetry, do not minimize flight distance during spring migration. Ibis.

[82] Bruderer B, Boldt A (2001). Flight characteristics of birds: I. radar measurements of speeds. Ibis.

[83] Lisovski S, Hahn S (2012). GeoLight - processing and analysing light-based geolocator data in R. Meth Ecol Evol.

[84] Kanamitsu M, Ebisuzaki W, Woollen J, Yang S-K, Hnilo JJ, Fiorino M, Potter GL (2002). NCEP-DOE AMIP-II reanalysis (R-2). Bull Am Meteorol Soc.

[85] Kemp MU, van Loon E, Shamoun-Baranes J, Bouten W (2012). RNCEP: global weather and climate data at your fingertips. Meth Ecol Evol.

[86] Schmaljohann H, Becker PJJ, Karaardic H, Liechti F, Naef-Daenzer B, Grande C (2011). Nocturnal exploratory flights, departure time, and direction in a migratory songbird. J Ornithol.

[87] Schmaljohann H, Liechti F, Bruderer B (2009). Trans-Sahara migrants select flight altitudes to minimize energy costs rather than water loss. Behav Ecol Sociobiol.

[88] Chelton DB, GFreeilich MH (2005). Scatterometer-based assessment of 10-m wind analyses from the operational ECMWF and NCEP numerical weather prediction models. Mon Weather Rev.

[89] Bromwich DH, Wang S-H (2005). Evaluation of the NCEP–NCAR and ECMWF 15- and 40-Yr reanalyses using rawinsonde data from two independent Arctic field experiments. Mon Weather Rev.

[90] Schmaljohann H, Bruderer B, Liechti F (2008). Sustained bird flights occur at temperatures beyond expected limits of water loss rates. Anim Behav.

[91] Mateos-Rodríguez M, Liechti F (2012). How do diurnal long-distance migrants select flight altitude in relation to wind?. Behav Ecol.

[92] Kemp MU, Shamoun-Baranes J, van Loon EE, McLaren JD, Dokter AM, Bouten W (2012). Quantifying flow-assistance and implications for movement research. J Theor Biol.

[93] Babak N. usdm: Uncertainty analysis for species distribution models. R package version 11–12. 2013.http://CRAN.R-project.org/package=usdm. Accessed 29 May 2015.

[94] Zuur AE, Irwin DE, Elphick CS (2010). A protocol for data exploration to avoid common statistical probelms. Meth Ecol Evol.

[95] Bates D, Mächler M, Bolker B, Walker S. lme4: Linear mixed-effects models using Eigen and S4. R package version 11–7. 2014. https://cran.r-project.org/web/packages/lme4/index.html. Accessed 29 May 2015.

[96] Korner-Nievergelt F, Roth T, von Felten S, Guélat J, Almasi B, Korner-Nievergelt P (2015). Bayesian data analysis in ecology using linear models with R, BUGS, and Stan.

[97] Forstmeier W, Wagenmakers E-J, Parker TH. Detecting and avoiding likely false-positive findings – a practical guide. Biol Rev. 2016:10.1111/brv.12315. 10.1111/brv.12315 27879038

[98] Gelman A, Hill J (2007). Data analysis using regression and multilevel/hierarchical models.

[99] Fox J (2003). Effect displays in R for generalised linear models. J Stat Softw.

[100] Fudickar AM, Wikelski M, Partecke J (2012). Tracking migratory songbirds: accuracy of light-level loggers (geolocators) in forest habitats. Meth Ecol Evol.

[101] Åkesson S, Walinder G, Karlsson L, Ehnbom S (2002). Nocturnal migratory flight initiation in reed warblers *Acrocephalus scirpaceus*: effect of wind on orientation and timing of migration. J Avian Biol.

[102] Dierschke V, Delingat J (2001). Stopover behaviour and departure decision of northern wheatears, *Oenanthe oenanthe*, facing different onward non-stop flight distances. Behav Ecol Sociobiol.

[103] Bairlein F, Eikenaar C, Schmaljohann H (2015). Routes to genes: unravelling the control of avian migration—an integrated approach using Northern Wheatear *Oenanthe oenanthe* as model organism. J Ornithol.

[104] Sapir N, Horvitz N, Wikelski M, Avissar R, Mahrer Y, Nathan R (2011). Migration by soaring or flapping: numerical atmospheric simulations reveal that turbulence kinetic energy dictates bee-eater flight mode. Proc R Soc Lond B.

[105] Fransson T (1998). Patterns of migratory fuelling in Whitethroats *Sylvia communis* in relation to departure. J Avian Biol.

[106] Tsvey A, Bulyuk VN, Kosarev V (2007). Influence of body condition and weather on departures of first-year European robins, *Erithacus rubecula*, from an autumn migratory stopover site. Behav Ecol Sociobiol.

[107] Richardson WJ (1978). Timing and amount of bird migration in relation to weather: a review. Oikos.

[108] Cohen EB, Pearson SM, Moore FR (2014). Effects of landscape composition and configuration on migrating songbirds: inference from an individual-based model. Ecol Appl.

[109] Cohen EB, Moore FR, Fischer RA (2012). Experimental evidence for the interplay of exogenous and endogenous factors on the movement ecolgy of a migrating songbird. PLoSONE.

[110] Panov IN, Chernetsov N (2010). Migratory strategy of bluethroats, *Luscinia svecica*, in eastern fennoscandia. Part 2: response to acoustic markers and habitat selection at stopover. Proc Zool Inst RAS.

[111] Bibby CJ, Green RE (1981). Autumn migration strategies of reed and sedge warblers. Ornis Scand.

[112] DeLuca WV, Woodworth BK, Rimmer CC, Marra PP, Taylor PD, McFarland KP, Mackenzie SA, Norris DR (2015). Transoceanic migration by a 12 g songbird. Biol Lett.

[113] Tøttrup AP, Klaassen RHG, Kristensen MW, Strandberg R, Vardanis Y, Lindström Å, Rahbek C, Alerstam T, Thorup K (2012). Drought in Africa caused delayed arrival of European songbirds. Science.

[114] Bairlein F (2002). How to get fat: nutritional mechanisms of seasonal fat accumulation in migratory songbirds. Naturwissenschaften.

[115] Bairlein F, Gwinner E (1994). Nutritional mechanisms and temporal control of migratory energy accumulation in birds. Ann Rev Nutr.

[116] Maggini I, Bairlein F (2010). Endogenous rhythms of seasonal migratory body mass changes and nocturnal restlessness in different populations of northern wheatear *Oenanthe oenanthe*. J Biol Rhyth.

[117] Eikenaar C, Tsvey A, Schmaljohann H (2015). Faster spring migration in northern wheatears is not explained by an endogenous seasonal difference in refueling rates. J Avian Biol.

[118] Charmantier A, Gienapp P (2014). Climate change and timing of avian breeding and migration: evolutionary versus plastic changes. Evol Appl.

[119] Charmantier A, McCleery RH, Cole LR, Perrins CM, Kruuk LEB, Sheldon BC (2008). Adaptive phenotypic plasticity in response to climate change in a wild bird population. Science.

[120] Newton I (2008). The migration ecology of birds.

[121] Easterling DR, Meehl GA, Parmesan C, Changnon SA, Karl TR, Mearns LO (2000). Climate extremes: observations, modeling, and impacts. Science.

[122] Gienapp P, Teplitsky C, Alho JS, Mills JA, Merilä J (2008). Climate change and evolution: disentangling environmental and genetic responses. Mol Ecol.

[123] Newson SE, Moran NJ, Musgrove AJ, Pearce-Higgins JW, Gillings S, Atkinson PW, Miller R, Grantham MJ, Baillie SR (2016). Long-term changes in the migration phenology of UK breeding birds detected by large-scale citizen science recording schemes. Ibis.

