# Supplementary material for: Flexible reaction norms to environmental variables along the migration route and the significance of stopover duration for total speed of migration in a songbird migrant
Source: Front Zool. 2017 Mar 20;14:17. doi: 10.1186/s12983-017-0203-3 (PMC5360013; doi:10.1186/s12983-017-0203-3)
Supplement: Additional file 1: — Individual data of birds, table. (DOCX 39 kb) [file 12983_2017_203_MOESM1_ESM.docx]

Individual data of birds tagged with a light-level geolocator about breeding locations, years of deployment, geolocator types [Biotrack (BT), Wareham, UK; British Antarctic Survey (BAS), Cambridge, UK; migrate technology (MT), Cambridge, U]. Leg-loop harness were used ([Rappole & Tipton 1990](#_ENREF_4)) and made with elastic O-rings (diameter: 27.5 mm, thickness: 1 mm) consisting of a silicone–rubber mixture (MVQ Arcus, Germany; <http://www.arcusshop.de/>). Number of false sunrise and false sunset events that were discarded and number of sunrises and sunsets that were adjusted were given. “old males” are older than 2^nd^ calendar year (cy) birds. “old females” are older than 1^st^ cy birds.

| Birds | Breeding location | Year of deployment | Sex and age at initial capture | Geolocator type (company) | Weight including harness [g] | Number of original twilight events | Number of discarded / adjusted sunrises and sunsets |
| --- | --- | --- | --- | --- | --- | --- | --- |
| 7902,  7910,  7916 | Eagle Summit | 2009,  2009,  2009 | 2^nd^ cy male,  old male,  old female | Mk10S (BAS) | 1.4,  1.4,  1.4 | 545,  573,  569 | 6/6,  8/1,  4/9 |
| B070 | Eagle Summit | 2013 | old male | ML6190 (BT) | 0.9 | 623 | 51/12 |
| E552,  E801,  E823 | Eagle Summit | 2013,  2013,  2013 | old male,  old male,  1^st^ yr male | Intigeo P65A9-11 (MT) | 0.9,  0.9,  0.9 | 579,  575,  577 | 7/11,  8/34,  4/7 |
| E553 | Toolik | 2013 | old male | Intigeo P65A9-11 (MT) | 0.9 | 565 | 5/19 |
