# Supplementary material for: Flexible reaction norms to environmental variables along the migration route and the significance of stopover duration for total speed of migration in a songbird migrant
Source: Front Zool. 2017 Mar 20;14:17. doi: 10.1186/s12983-017-0203-3 (PMC5360013; doi:10.1186/s12983-017-0203-3)

**Recapture probability of tagged Northern Wheatears**

The recapture probability differed between the years (2010: 5/30 = 16% and 2014: 8/240 = 3%). Northern Wheatears tolerated well carrying light-level geolocators ([Bairlein *et al.* 2012](#_ENREF_2); [Schmaljohann *et al.* 2012a](#_ENREF_5); [Schmaljohann *et al.* 2012b](#_ENREF_6)). As light-level geolocators were smaller and lighter in 2013 than in 2009 (Table S1), we did not suspect a stronger influence of the tags in 2013. During a past compatibility test, we attached dummy geolocators (1.4 g) to 12 birds of our indoor breeding population for 6 months in 2008, and the birds could freely fly. They adjusted their body mass seasonally to high values during migration but low values otherwise. Leg-loop harnesses fit well, regardless of these natural changes in body mass. The birds did not differ in their flight behaviour from the control birds, and they did not show any damage to their feathers or skin ([Schmaljohann *et al.* 2012b](#_ENREF_6)). The same was true for the free-flying tagged Northern Wheatears of the Rhineland-Palatinate population in Germany. We could find no apparent difference in the clutch size between pairs with males carrying geolocators (5.8±0.45 eggs, n=5) and pairs with males without geolocators (5.2±1.0 eggs, n=74; Wilcoxon test: p=0.089) in 2010, and no differences were observed in the first brood’s hatching success between males carrying geolocators (97 % , 28 chicks out of 29 eggs, n=5) and males without geolocators (91 %, 346 chicks out of 382 eggs, n=74; X2= 0.013, p=0.91; ([Schmaljohann *et al.* 2012a](#_ENREF_5))). Based on these studies, Northern Wheatears were assumed to generally tolerate carrying light-level geolocators well. However, geolocators may affect the seasonal timing of movements and breeding success in some Northern Wheatears ([Arlt *et al.* 2013](#_ENREF_1)). The low recapture success in 2014 was likely explained by extremely low temperatures and consistent heavy rainfall in the 2014 summer, which was the coldest and wettest summer within living memory (Tables S2, 3, Figure S1). As such conditions significantly reduce breeding success and parental survival in Northern Wheatears ([Öberg *et al.* 2015](#_ENREF_3)), many Northern Wheatears did not attempt to breed and were not territorial in Alaska in the summer of 2014 (own personal observations). This phenomenon resulted in a dramatically decreased chance of resighting and catching tagged wheatears. The most likely explanation for the low recapture rate in 2014 was that the unfavourable weather conditions prevented the birds from breeding in the 2014 summer.

**Figure S1** Daily maximum temperature [°F] at Fairbanks from 1^st^ of May to 20^th^ of July 2014 over season. Data are shown in comparison to the mean and standard deviation of temperature data from 1981 to 2010. Figure was downloaded from <http://ak-wx.blogspot.de/2014/07/cool-summer-days.html> on 22.04.2015.


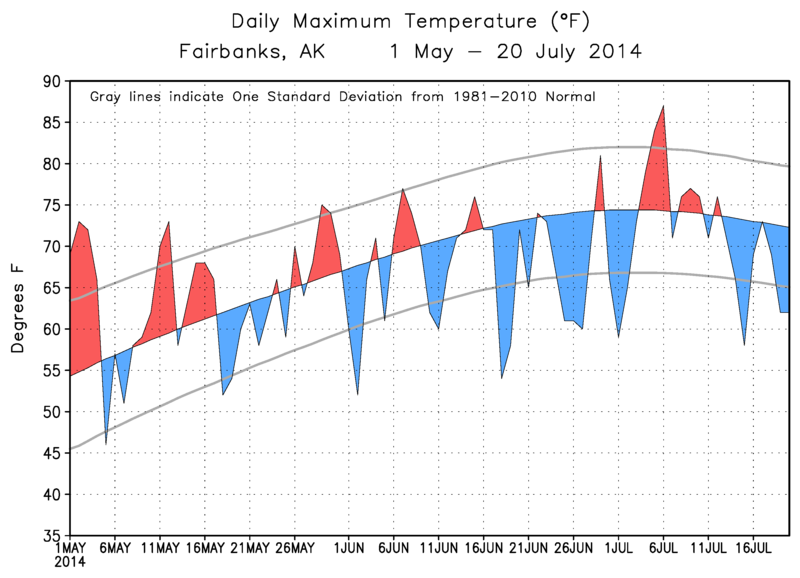


**Table S1** Temperature (temp.) and precipitation (prec.) at Eagle Summit and at Toolik in the summers 2013 and 2014. For each year data from 21^st^ of May until 30^th^ of June (n_days_ = 41) were considered, as Northern Wheatears arrive in their Alaskan breeding areas in the last decade of May and start breeding in June (own pers. obs.). At Eagle Summit and the Toolik Field Station both parameters were obtained from a local weather stations (Eagle Summit: U.S. Department of Agriculture, Natural Resources Conservation Service, site number: 960, location: 65.49° N, 145.41° W, 1113 m above sea level [TEMP DATA ES]; Toolik Field Station, 68.63°N, 149. °W, 720 m above sea level [TEMP DATA TOLLIK]). Temperature was considered as a daily mean based on 24 hourly measurements per day and precipitation data as total amount per day. To assess for differences in precipitation between years, we run Wilcoxon signed rank tests, as data were not normally distributed.

| **Area** | **Year** | **Daily mean temp. [°C]**  mean ± s.d. | t-test | **Daily mean prec. [mm]**  mean ± s.d. | Wilcoxon test | **Number of days with rain** | Wilcoxon test | **Total prec. [mm]** |
| --- | --- | --- | --- | --- | --- | --- | --- | --- |
| **Eagle Summit** | 2013 | 11.9 ± 4.2 | t=5.8, df=74.8, p<0.0001 | 0.93 ± 3.54 | W=653,  p=0.019 | 5 | W=615,  p=0.0058 | 38.1 |
|  | 2014 | 7.1 ± 3.2 |  | 1.61 ± 2.71 |  | 16 |  | 66.0 |
| **Toolik** | 2013 | 7.3 ± 6.4 | t=2.0, df=76.6, p=0.046 | 1.41 ± 2.53 | W=709  P=0.204 | 21 | W=738  P=0.27 | 57.8 |
|  | 2014 | 4.7 ± 5.2 |  | 1.98 ± 3.12 |  | 26 |  | 81 |

**Table S2** Total precipitation [inch] at Fairbanks for different time periods of 2014. Data are shown in comparison with other years that likewise showed high precipitation.Table was downloaded from <http://ak-wx.blogspot.de/2014/09/september-precipitation-days.html> on 22.04.2015.


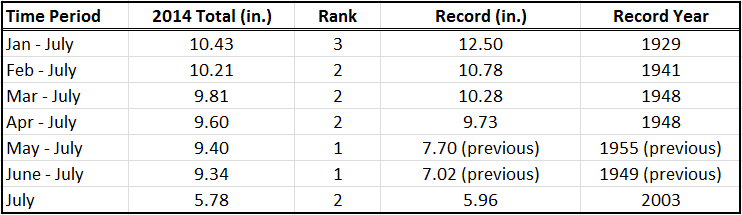

Supplement: Additional file 2: — Recapture probability of tagged Northern Wheatears, documentation. (DOCX 159 kb) [file 12983_2017_203_MOESM2_ESM.docx]
