# Supplementary material for: Flexible reaction norms to environmental variables along the migration route and the significance of stopover duration for total speed of migration in a songbird migrant
Source: Front Zool. 2017 Mar 20;14:17. doi: 10.1186/s12983-017-0203-3 (PMC5360013; doi:10.1186/s12983-017-0203-3)
Supplement: Additional file 3: — R code for analyzing light-level geolocation data, documentation. (DOCX 186 kb) [file 12983_2017_203_MOESM3_ESM.docx]

### Appendix - R code for analyzing light-level geolocation data

###

**Heiko Schmaljohann & Simeon Lisovski**

**Content:**
1. General stuff
2. Bird data
3. Estimating twilights
4. Simple threshold estimates
5. Essie model
6. Estell model
7. Defining migratory schedule

### 1. General stuff

## defining work directory
wd <- "your work directory"

# set R to GMT
Sys.setenv(tz="GMT")

# required packages
library(GeoLight)
library(SGAT)
library(BAStag)
library(maptools)

# loading world data
data(wrld_simpl)

### 2. Bird data

# individual bird id, location of breeding area, and date of deployment
bird <- data.frame(# bird id
 ID = c("7902","7910","7916", "B070",
 "E552", "E553", "E801", "E823"),
 # longitude of breeding area (minus is West)
 lon.breed = c(-145.389167, -145.3731944, -145.3852778, -145.387000,
 -145.385083, -149.522676, -145.392139, -146.017583),
 # latitude of breeding area
 lat.breed = c(65.4988056, 65.5037222, 65.4885556, 65.497028,
 65.499944, 68.628406, 65.488528, 65.397083),
 # date of deployment
 tagged = c(as.POSIXct("2009-06-19", tz = "GMT"),
 as.POSIXct("2009-06-25", tz = "GMT"),
 as.POSIXct("2009-06-26", tz = "GMT"),
 as.POSIXct("2013-06-29", tz = "GMT"),
 as.POSIXct("2013-06-27", tz = "GMT"),
 as.POSIXct("2013-06-18", tz = "UTC"),
 as.POSIXct("2013-07-01", tz = "GMT"),
 as.POSIXct("2013-05-06", tz = "GMT")),
 # manufacturer of light-level geolocator:
 # biotrack = Biotrack and British Antartic Survey, identical data processing
 # MigTech = migrate Technology
 manufacturer = c(rep("biotrack",4),rep("MigTech",4)))

# selecting bird for analysis and setting corresponding variable
 # the script is run here for bird "E552"
ID <- "E552"
lon.calib <- bird$lon.breed[bird$ID == ID]
lat.calib <- bird$lat.breed[bird$ID == ID]
tagged.date <- bird$tagged[bird$ID == ID]

### 3. Estimating twilights

**Data import**

# depending on the manucfacturer either the frist paragraph or the second needs to be run

# light data of birds "7902", "7910", "7916", and "B070" are in "lig" files
#d.lux <- read.table(paste(wd,"/data/", ID, ".lig", sep=""), header = FALSE, skip = 25,
# sep=",",
# col.names = c("check", "Date", "no_interest", "Light"),
# colClasses = c("character", "character", "character", "numeric"))
#d.lux$Date <- as.POSIXct(strptime(d.lux$Date, "%d/%m/%y %H:%M:%S", tz = "GMT"))
#d.lux <- subset(d.lux, Date>=tagged.date)
#str(d.lux)

# or

# light data of the other birds are in "lux" files"
d.lux <- read.table(paste(wd,"/data/", ID, ".lux", sep=""), header = FALSE, skip = 25,
 col.names = c("Date","Time", "Light"),
 colClasses = c("character", "character", "numeric"))
d.lux$Date <- as.POSIXct(strptime(paste(d.lux$Date, d.lux$Time), "%d/%m/%Y %H:%M:%S", tz = "GMT"))
d.lux$Light <- d.lux$Light
d.lux <- subset(d.lux, Date>=tagged.date)
str(d.lux)

## 'data.frame': 105470 obs. of 3 variables:
## $ Date : POSIXct, format: "2013-06-27 00:04:03" "2013-06-27 00:09:03" ...
## $ Time : chr "00:04:03" "00:09:03" "00:14:03" "00:19:03" ...
## $ Light: num 1.14 1.14 1.14 1.14 1.14 ...

**Plotting light-data and subset of light-data over date**

# defining offset for convenient use of light-data on y axis
offset <- 6

opar <- par(mfrow = c(1, 2), mar = c(4,4,1,1))
lightImage(d.lux, offset = offset)
# light intensity is recorded differently depending on the type of the light-level geolocator
# Biotrack geolocators use an abritrary unit, light intensity increases from 0 to 64 (max)
# Migrate Technoloy geolocators record light intensity in lux, unit of illuminance, increasing from 0 to 74,000 lux
plot(d.lux$Light[20000:25000], type = "o", cex = 0.5, pch = 16, ylab = "log(Light) (lux)", xlab = "Date")


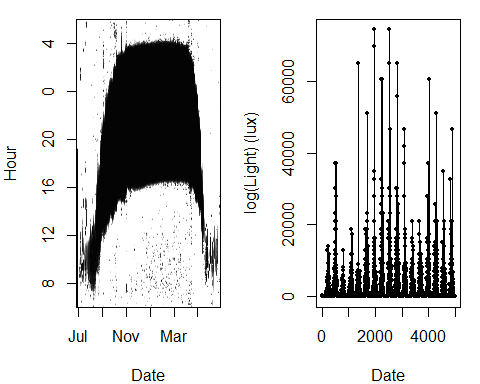


par(opar)

**Set threshold**

# Threshold for simple threshold method
threshold <- 1.35

**Zenith has to be choosen individually**

# Estimating zenith
 # 1. estimates individual zenith values visually by plotting light intensity over zenith
 t.diff <- bird$tagged[bird$ID == ID] - min(d.lux$Date)
 # limit calibration data until date set above for specific geolocator
 d.calib <- subset(d.lux,Date < min(Date) + 40*24*60*60 & Date > min(Date) + 7*24*60*60)
 max(d.calib$Date)
 opar <- par(mfrow = c(1, 2), mar = rep(4, 4))
 lightImage(d.calib, offset = offset)
 thresholdCalibrate(d.calib, bird[bird$ID == ID, "lon.breed"],bird[bird$ID == ID, "lat.breed"],
 max.adjust = T, xlim = c(88, 99), ylim = c(0, 15), pch= 16)
 abline(v=97.5,col="orange",lwd=2)
 par(opar)

 # 2. select and define zenith for further calculations; this may differ from the estimated zenith above, if diagnostic plots run below call for zenith adjustment
 bird$zenith <- c(0)
 bird$zenith[bird$ID == ID] <- 97.5
 # 7902: zenith used 97
 # 7910: zenith used 97
 # 7916: zenith used 97
 # B070: zenith used 93.7
 # E552: zenith used 97.5
 # E553: zenith used 97.5
 # E801: zenith used 97.0
 # E823: zenith used 97.82

i.zenith <- bird$zenith[bird$ID == ID]
i.zenith

**Twilight times**

Twilights were defined with the preprocess.light() function.
The process has four stages:

1. The subset of data is selected (left click=start, right click=end)
2. Twilight search (left click = search point, right click = prevent search)
3. Insert missing twilights (left click = sunset, right click = sunrise, 0-9 sets markers)
4. Edit individual twilight (left click = select time, right click = delete, 0-9 sets markers)

In stages 3 and 4, setting a marker with a known location sets the twilight time to the time of twilight for that location.

In stage 4 the first two days were marked as beeing recorded at the individual breeding site. Also in stage 4 some twilights were adjusted manually, see individual output of changes made in twilights (Table A2 of the supplemental materials). The preprocess.light() function calls an interactive procedure so that the corresponding images cannot be displayed here. For further information about this function see the corresponding help file by typing "?preprocess.light" in the R console.

Consider that Alaskan Northern Wheatears have been shown to resume migration shortly after sunset (Schmaljohann et al. 2013). As birds can fly several kilometers above ground level, it seems likely that some of the Alaskan Northern Wheatears experienced some light after departure. These short periods of light occurring after local sunset were not considered as sunrise and/or sunset events. They occurred mostly at the beginning of autumn migration. This is also the reason why some sunrise and sunset events were adjusted by maximal about 30 minutes (Table S1 of the supplemental materials).

twl <- preprocessLight(d.lux, threshold, offset = offset, zenith = i.zenith, lmax = 35,
 fixed = matrix(c(lon.calib, lat.calib), ncol = 2))
# adjust twilight estimates
# depending on the type of light-level geolocator the sampling interval needs to be defined
# # Biotrack (former British Antarctic Sruvey) = 600 [s]
# # Migrate Technology = 300 [s]
# this is automatically considered here in the correct way
 twl <- twilightAdjust(twilights = twl, interval = ifelse(bird$manufacturer[bird$ID==ID] == "biotrack", 600, 300))
 write.csv(twl, paste(wd, "/results/", ID, "_twl.csv", sep=""), row.names = FALSE)

twl <- read.csv(paste(wd, "/results/", ID, "_twl.csv", sep=""))
str(twl)

## 'data.frame': 578 obs. of 7 variables:
## $ Twilight : Factor w/ 578 levels "2013-07-31 09:53:03",..: 1 2 3 4 5 6 7 8 9 10 ...
## $ Rise : logi FALSE TRUE FALSE TRUE FALSE TRUE ...
## $ Deleted : logi TRUE TRUE FALSE FALSE TRUE TRUE ...
## $ Marker : int 0 0 0 0 0 0 0 0 0 0 ...
## $ Inserted : logi FALSE FALSE FALSE FALSE FALSE FALSE ...
## $ Twilight3: Factor w/ 578 levels "2013-07-31 09:58:03",..: 1 2 3 4 5 6 7 8 9 10 ...
## $ Marker3 : int 0 0 0 0 0 0 0 0 0 0 ...

twl$Twilight <- as.POSIXct(twl$Twilight,"GMT")
twl$Twilight3 <- as.POSIXct(twl$Twilight3,"GMT")

lightImage(d.lux, offset = offset)
tsimagePoints(twl$Twilight[twl$Deleted == F], offset = offset, pch = 16,
 col = ifelse(twl$Rise[twl$Deleted == F], "dodgerblue", "firebrick"))


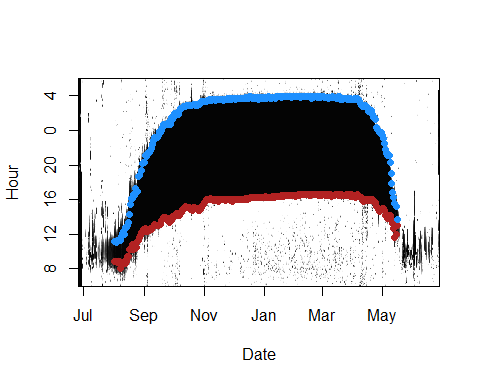


This plot shows the entire recorded light data. Black is night. Blue dots indicate sunrise events and red dots sunset events.

###4. Simple threshold estimates

**Bird's location are here initially estimated with the "simple" threshold approach.**

First, a certain tolerance on the sine of the solar declination needs to be defined. This is done by selecting such a value that minimize variation during equinoxes.

# select tolerance on the sine of the solar declination
#for(i in 1:39)
#{
# t.tol <- seq(0.01,0.2,0.005)[i]
# path <- thresholdPath(twl$Twilight[twl$Deleted==F], twl$Rise[twl$Deleted==F], zenith = i.zenith, tol = t.tol)
# # plotting longitude estimates and latitude estimates over season
# opar <- par(mfrow = c(2, 1), mar = c(3,5,1,1))
# plot(path$time, path$x[, 1], type = "b", pch = 16, cex = 0.5, ylab = "Lon", main = t.tol)
# plot(path$time, path$x[, 2], type = "b", pch = 16, cex = 0.5, ylab = "Lat", main = t.tol)
# abline(h = lat.calib, col = "firebrick", lty = 2)
# par(opar)
# }

# for illustration purposes one figure for E552 is shown here
path <- thresholdPath(twl$Twilight[twl$Deleted == F], twl$Rise[twl$Deleted == F], zenith = 97.5, tol = 0.15)
 # plotting longitude estimates and latitude estimates over season
 opar <- par(mfrow = c(2, 1), mar = c(3,5,1,1))
 plot(path$time, path$x[, 1], type = "b", pch = 16, cex = 0.5, ylab = "Lon", main = "0.15")
 plot(path$time, path$x[, 2], type = "b", pch = 16, cex = 0.5, ylab = "Lat", main = "0.15")
 abline(h = lat.calib, col = "firebrick", lty = 2)


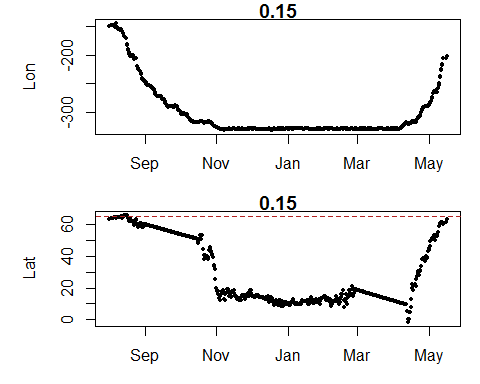


# select individual value
bird$tol <- c(0)
bird$tol[bird$ID == ID] <- 0.15 # for bird E552
 # indidivually selected values
 #7902: tol = 0.085
 #7910: tol = 0.125
 #7916: tol = 0.13
 #B070: tol = 0.18
 #E552: tol = 0.15
 #E553: tol = 0.075
 #E801: tol = 0.155
 #E823: tol = 0.135
tol <- bird$tol[bird$ID==ID]

**Initial track based on simple threshold estimates**

# it is sufficent to fix only the first position to the breeding area
fixedx <- c(TRUE, rep(FALSE, nrow(twl[twl$Deleted == F,])-1))

path$x[1,1] <- lon.calib
path$x[1,2] <- lat.calib

opar <- par(mfrow = c(1,1))
plot(path$x, type = "n")
plot(wrld_simpl, add = T, col = "grey95")
plot(elide(wrld_simpl, shift = c(-360, 0)), add = T, col = "grey95")
lines(path$x, col = "blue")
points(path$x, pch = 16, cex = 0.3, col = "blue")
points(path$x[1,1], path$x[1,2], pch = 1, cex = 2, col = "green", lwd=3)
box()


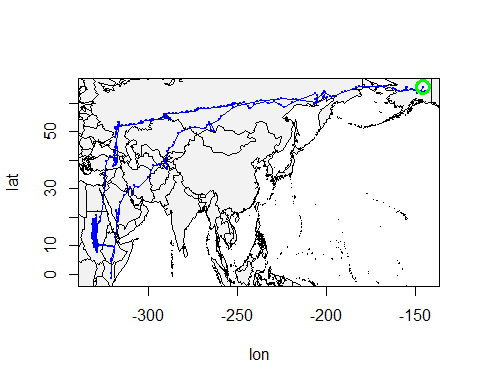


par(opar)

### 5. Essie Model

With the Essie Model the first approximate tracks are produced that are used further below to initialize the full analysis (see 6. Estelle model). A specified log-normal distribution for the twilight errors and a gamma distribution out of which bird's travel speed is choosen are defined. The boundaries of the grid ("xlim" and "ylim") in which the birds is supposed to move are estimated.

**Model parameters**

Set the model parameters controlling the log-normal distribution of twilight errors and the gamma speed distribution. For both conservative distributions were assumed.

# twilight errors
alpha <- c(2.2,1.0)
# speed distribution
beta <- c(0.7, 0.05)

opar <- par(mfrow = c(1,2))
m <- seq(0, 60, length = 80)
plot(m, dlnorm(m, alpha[1], alpha[2]), type="l", xlab = "Twilight Error (mins)", ylim = c(0, 0.2),
 lwd = 2, main="twilight error distribution")

kmh <- 1:90
plot(kmh, dgamma(kmh, beta[1], beta[2]), type = "l", col = "orange", lwd = 2,
 main="speed distribution" )


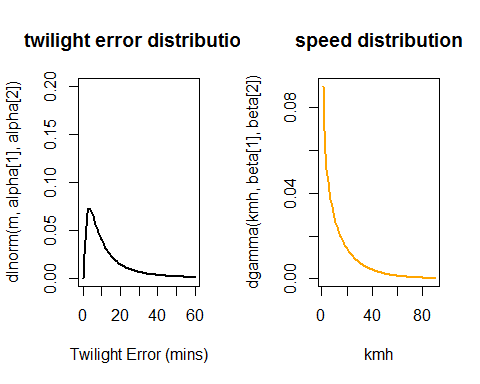


par(opar)

**Area of potential occurrence**

Here the land mask is defined. Northern Wheatears are small songbirds that cannot rest on water. Therefore, a priori low probability was given for locations on water. However, it was important to close the gap between Russia and the US, i.e., the Bering Strait, because otherwise the binary probability of the land mask has problems letting the birds pass this short ecological barrier. The bounding box defines the geografical limit of bird's whereabouts.

Bounding box

# define bounding box, i.e., area where bird could potenially be
xlim <- c(floor(min(path$x[,1])) - 10, ceiling(max(path$x[,1])) + 10)
ylim <- c(max(floor(min(path$x[,2])) - 10, - 85), min(ceiling(max(path$x[,2])) + 10, 85))

Spatial raster

n <- 1
grid <- raster(nrows = n*diff(ylim),ncols = n*diff(xlim),
 xmn = xlim[1],xmx = xlim[2],ymn = ylim[1],ymx = ylim[2],
 crs = proj4string(wrld_simpl))
grid <- cover(rasterize(elide(wrld_simpl,shift = c(-360, 0)), grid, 1, silent = TRUE),
rasterize(wrld_simpl, grid, 1, silent = TRUE))
## mask must be non-zero for sea
grid <- !is.na(grid)
grid[][coordinates(grid)[,1] > -200 & coordinates(grid)[,2] > 62.5 & coordinates(grid)[,2]<69] <- 1

opar <- par(mfrow = c(1, 1))
plot(grid)


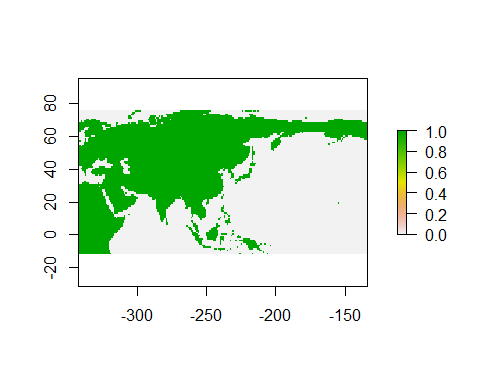


par(opar)

**Model Fit**

The Essie Model has a forward and backward phase. The final estimate of the model is then a combination of these two phases which is considered in the Estelle Model, see below.

fixed <- fixedx > 0
x0 <- as.matrix(path$x)

model <- essieThresholdModel(twl$Twilight[twl$Deleted == F], twl$Rise[twl$Deleted == F],
 alpha = alpha, beta = beta,
 x0 = x0, fixed = fixed, zenith = 97.5)

fit.essie <- essie(model, grid, epsilon1 = 1.0E-1, epsilon2 = 0.0)


opar <- par(mfrow = c(2, 2), mar=rep(2,4))
plot(fit.essie$grid, legend = FALSE, col = c("honeydew3", "white"), main = "Track")
lines(essieMean(fit.essie)$x, type = "l", col = "steelblue")
plot(fit.essie$grid, legend=FALSE, col = c("honeydew3", "white"), main = "Forward")
lines(essieMean(fit.essie, type="forward")$x, type = "l", col = "steelblue")
plot(fit.essie$grid, legend = FALSE, col = c("honeydew3", "white"), main = "Backward")
lines(essieMean(fit.essie, type = "backward")$x, type = "l", col = "steelblue")
par(opar)


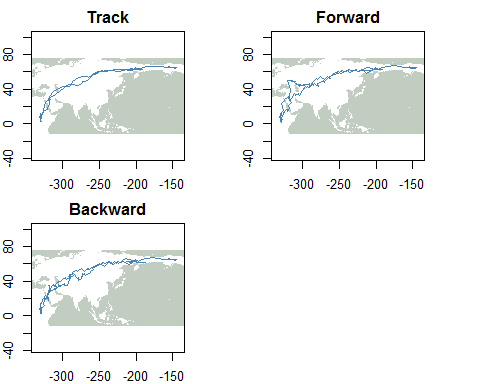


If the initial location estimates are resonable, as here, one can proceeed with the estelle process. If these initial location estimates vary strongly between the two phases, a different zenith angle and/or tolerance values have to be chosed. This is a trail and error procedure.

###6. Estelle model

**Spatial land mask**

As Alaskan Northern Wheatears have to cross the Bering Strait between western Alaska and Chukotcha, the Caspian Sea, and parts of the Red Sea, location estimates at sea should not be entirely prohibited. In the log.prior function, the probabilities are relative measures. The land mask indicates when location estimates of the path are at sea or fall out of the bounding box as defined above, see "xlim" and "ylim" above and use this to define a log prior that is applied within the modelling.

# mask is required; the corresponding r file is provided as electronic supplemental material
source(paste0(wd,"mask.r")

is.sea <- land.mask(xlim=xlim + c(+5, -5),ylim=ylim+c(-5, 4),n=4,land=F)

log.prior <- function(p) {
 f <- is.sea(p)
 ifelse(!is.na(f) & f,log(1),log(10))

}

log.prior.x <- log.prior
log.prior.z <- log.prior

**Two speed distributions**

Alaskan Northern Wheatears cover during each autumn and spring migration season about 15,000 km within three months in autumn and about two months in spring. These birds reach, therefore, relatively high daily travel distances. To allow for these we apply here two different speed distributions, a "slow" one that is characteristic for small songbird migrants in general and a "fast" one especially adjusted for the Alaskan Northern Wheatear. Later the "slow" speed distribution was applied to stationary sites and the "fast" speed distribution to migratory periods, see below for details. Please, consider that this is step can be possibly ignored in other species.

# slow speed (red in figure) is defined with shape = 5 and scale = 3
# fast speed (blue in figure) is defined with shape = 12 and scale = 0.3
beta <- matrix(c(5, 3, 12, 0.3), 2, 2, byrow = T)

opar <- par(mfrow=c(1,1))
matplot(kmh, cbind(dgamma(kmh, beta[1, 1], beta[1, 2]),
 dgamma(kmh, beta[2, 1], beta[2, 2])),
 type = "l", col = c("red", "blue"), lty = 1, lwd = 2,
 ylab = "", xlab = "Flight speed [km/h]")
text(x = 45, y = 0.05, labels = "fast", col = "blue")
text(x = 10, y = 0.05, labels = "slow", col = "red")


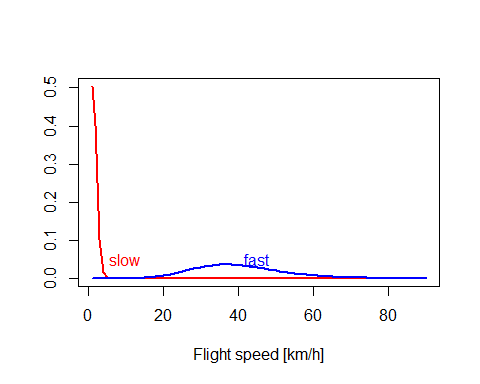


par(opar)

**Provide the speed states**

The changeLight function from the R Package GeoLight is used to set the speed states.

# transform the data to fit GeoLight requirements
tFirst <- twl[twl$Deleted == F, 1][-nrow(twl[twl$Deleted == F,])]
tSecond <- twl[twl$Deleted == F, 1][-1]
type <- ifelse(twl[twl$Deleted == F, 2], 1, 2)[-nrow(twl[twl$Deleted == F,])]

cL <- changeLight(tFirst = tFirst, tSecond = tSecond, type = type, quantile = 0.70, days = 1, summary = F)


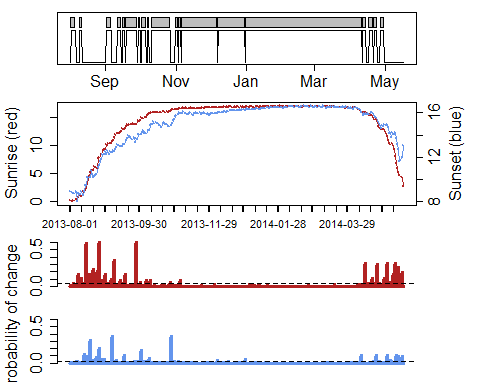


# The choice of the quantile is subjective. We set the quantile here for all birds constant to 0.7. For details see Lisovski & Hahn (2012).
par(mfrow=c(1,1))

Here we consider the initially estimated migration schedule when modelling bird's location.

x0 <- essieMode(fit.essie)$x
z0 <- trackMidpts(x0)

## setting beta accoridng to the changeLight output.
beta.cL <- matrix(c(ifelse(cL$site == 0, beta[2, 1], beta[1, 1]),
 ifelse(cL$site == 0, beta[2, 2], beta[1, 2])), ncol = 2)


model <- thresholdModel(na.omit(twl$Twilight[twl$Deleted == F]),na.omit(twl$Rise[twl$Deleted == F]),
 twilight.model = "ModifiedLogNormal",
 alpha = alpha, beta = beta.cL,
 logp.x = log.prior.x, logp.z = log.prior.z,
 x0 = x0, z0 = z0, zenith = 97.5, fixedx = fixedx)

x.proposal <- mvnorm(S = diag(c(0.005, 0.005)), n = nrow(x0))
z.proposal <- mvnorm(S = diag(c(0.005, 0.005)), n = nrow(z0))

fit <- estelleMetropolis(model, proposal.x = x.proposal, proposal.z = z.proposal,
 iters = 50, thin = 20, chains = 2)

Tuning

x0 <- chainLast(fit$x)
z0 <- chainLast(fit$z)
model <- thresholdModel(na.omit(twl$Twilight[twl$Deleted == F]), na.omit(twl$Rise[twl$Deleted == F]),
 twilight.model = "LogNormal",
 alpha = alpha, beta = beta,
 logp.x = log.prior.x, logp.z = log.prior.z,
 x0 = x0, z0 = z0, zenith = 97.5, fixedx = fixedx)

proposal.x <- mvnorm(S = diag(c(0.005, 0.005)), n = nlocation(x0))
proposal.z <- mvnorm(S = diag(c(0.005, 0.005)), n = nlocation(z0))

fit <- estelleMetropolis(model, proposal.x, proposal.z,
 iters = 300, thin = 20, chains = 2)

for(k in 1:3) {
 proposal.x <- mvnorm(chainCov(fit$x), s = 0.2)
 proposal.z <- mvnorm(chainCov(fit$z), s = 0.2)
 fit <- estelleMetropolis(model, proposal.x, proposal.z,
 x0 = chainLast(fit$x),
 z0 = chainLast(fit$z),
 iters = 300, thin = 20, chains = 2)
}

Final run

proposal.x <- mvnorm(chainCov(fit$x) , s = 0.25)
proposal.z <- mvnorm(chainCov(fit$z) , s = 0.25)
fit <- estelleMetropolis(model, proposal.x, proposal.z,
 x0 = chainLast(fit$x),
 z0 = chainLast(fit$z),
 iters = 4000, thin = 20, chains = 4)

# plot final track
fixedz <- fixedx[-length(fixedx)] > 0 & fixedx[-length(fixedx)] == fixedx[-1]
dt <- ifelse(fixedz, 0, diff(as.numeric(model$time)/3600))
im <- locationImage(fit$z, xlim = xlim, ylim = ylim, nx = 4 * diff(xlim), ny = 4 * diff(ylim), weight = dt)
plot(wrld_simpl, xlim = xlim, ylim = ylim, col = "grey80", border = "grey50")
plot(elide(wrld_simpl, shift = c(-360, 0)), xlim = xlim, ylim = ylim, add = T, col = "grey80", border = "grey50")
image(im$x, im$y, im$W, xlab = "Lon", ylab = "Lat", cex.axis = 0.7, add = T, col = c("transparent", rev(heat.colors(200))))
xm <- locationMean(fit$x)
lines(xm, col = "cornflowerblue")
points(xm, pch = 16, cex = 0.8, col = "cornflowerblue")
box()


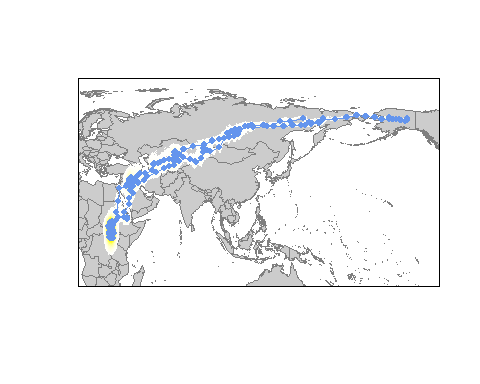


## save the fit
 save(fit, file = paste0(wd, "/results/", ID, ".track.RData"))

## save twilight data
 write.csv(twl, paste(wd, "/results/", ID, "_twl.csv", sep=""), row.names = FALSE)

 ## r VersionInfo,results='asis',echo=FALSE
 cat(R.version.string,"\n",
 "SGAT: ",as.character(packageVersion("SGAT")),"\n",
 "BAStag: ",as.character(packageVersion("BAStag")),sep="")

### 7. Defining migratory schedule

First, the twilight times are recalculated.

twl.back <- data.frame(Twilight = twilight(twl$Twilight[twl$Deleted == F], xm[,1], xm[,2],
 twl$Rise[twl$Deleted == F], zenith = 97.5),
 Rise = twl$Rise[twl$Deleted == F])
twl.back <- twilightAdjust(twl.back,
 -ifelse(bird$manufacturer[bird$ID==ID] == "biotrack", 600, 300))
opar <- par(mar = c(5,5,1,1))
lightImage(d.lux, offset = offset)
tsimagePoints(twl.back$Twilight, offset = offset, pch = 16, cex = 0.8,
 col = ifelse(twl.back$Rise, "dodgerblue", "firebrick"))


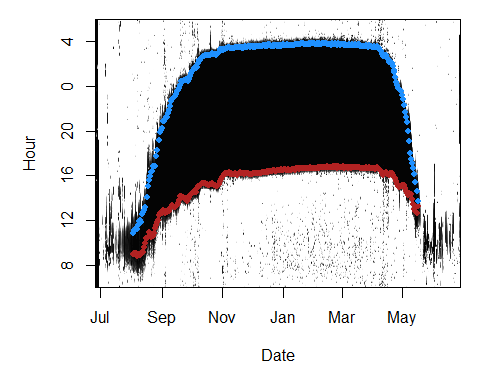


par(opar)

Second, twilight data is transformed to match GeoLight requirements and run changeLight to get migration schedule.

t.tFirst <- twl.back[-nrow(twl.back), 1]
t.tSecond <- twl.back[-1, 1]
t.type <- ifelse(twl.back[,2], 1, 2)[-nrow(twl.back)]

cL <- changeLight(tFirst = t.tFirst[-c(1,2)], tSecond = t.tSecond[-c(1,2)], type = t.type[-c(1,2)],
 quantile = 0.75, days = 2, summary = T)


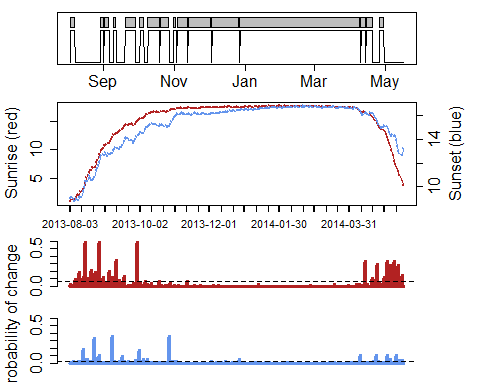


##
## Probability threshold(s):
##
## Sunrise: 0.07114 Sunset: 0.02361
##
##
## Migration schedule table:
##
## Site Arrival Departure Days P.start
## 1 a 2013-08-03 22:00:07 2013-08-07 10:00:05 3.5 0.029166667
## 2 b 2013-08-29 16:10:35 2013-08-31 16:29:43 2.0 0.015977961
## 3 c 2013-09-01 16:46:03 2013-09-05 17:02:17 4.0 0.015977961
## 4 d 2013-09-09 17:52:26 2013-09-11 18:07:44 2.0 0.014583333
## 5 e 2013-09-19 19:16:52 2013-09-28 07:20:12 8.5 0.000000000
## 6 f 2013-10-02 07:59:49 2013-10-05 08:04:45 3.0 0.027777778
## 7 g 2013-10-09 08:44:48 2013-10-18 20:57:23 9.5 0.029333333
## 8 h 2013-10-20 03:14:52 2013-10-27 09:01:34 7.2 0.000000000
## 9 i 2013-10-31 09:33:18 2013-11-02 09:41:35 2.0 0.000000000
## 10 j 2013-11-03 09:45:56 2013-11-11 21:46:01 8.5 0.005555556
## 11 k 2013-11-12 21:49:10 2013-12-02 09:50:57 19.5 0.000000000
## 12 l 2013-12-03 09:52:05 2013-12-26 10:01:16 23.0 0.000000000
## 13 m 2013-12-27 10:04:22 2014-04-09 09:45:30 103.0 0.000000000
## 14 n 2014-04-10 09:31:19 2014-04-13 21:28:58 3.5 0.007000000
## 15 o 2014-04-14 21:21:39 2014-04-19 21:09:14 5.0 0.005859375
## 16 p 2014-04-26 07:34:51 2014-04-29 19:19:23 3.5 0.057407407
## P.end Days.1 P.start.1
## 1 0.013541667 3.5 0.029166667
## 2 0.008333333 2.0 0.015977961
## 3 0.005729167 4.0 0.015977961
## 4 0.008333333 2.0 0.014583333
## 5 0.036570248 8.5 0.000000000
## 6 0.009259259 3.0 0.027777778
## 7 0.006122449 9.5 0.029333333
## 8 0.012500000 7.2 0.000000000
## 9 0.000000000 2.0 0.000000000
## 10 0.000000000 8.5 0.005555556
## 11 0.000000000 19.5 0.000000000
## 12 0.000000000 23.0 0.000000000
## 13 0.022110086 103.0 0.000000000
## 14 0.005859375 3.5 0.007000000
## 15 0.006481481 5.0 0.005859375
## 16 0.014115646 3.5 0.057407407

# "quantile" and "days" were set to 0.75 and 2 for all birds

Third, estimated sites are merged based on the distance between consequtive sites.

# getting mean location estimates per twilight event from the model (fit)
d.track <- locationSummary(fit$x)
d.track$time <- fit$model$time

# merging sites when closer than threshold distance
# fun.mergeSites is required; the corresponding r file is provided as electronic supplemental material
source(paste0(wd,"/paper/fun.mergeSites.r"))
mS <- fun.mergeSites(datetime = d.track$time[-c(1,2,nrow(d.track))],
 matrix(c(d.track$Lon.mean[-c(1,2,nrow(d.track))], d.track$Lat.mean[-c(1,2,nrow(d.track))]), ncol = 2, byrow = F),
 site = cL$site, threshold = 200, plot = T)
# In this case all sites were further away than 200 km. Therefore, no sites were merged.

## save the migration schedule and mering sites results
 save(cL, file=paste0(paste0(wd,"/results/",ID,".mig.RData")))
 save(mS, file=paste0(paste0(wd,"/results/",ID,".mSite.RData")))
