# Supplementary material for: Flexible reaction norms to environmental variables along the migration route and the significance of stopover duration for total speed of migration in a songbird migrant
Source: Front Zool. 2017 Mar 20;14:17. doi: 10.1186/s12983-017-0203-3 (PMC5360013; doi:10.1186/s12983-017-0203-3)
Supplement: Additional file 4: — R code for analyzing phenotypic response to environmental cues, documentation. (DOCX 147 kb) [file 12983_2017_203_MOESM4_ESM.docx]

### Appendix - R code for analyzing phenotypic response to environmental cues

###

**Heiko Schmaljohann**

**Content:** 1. General stuff 2. Aggregating data 3. Analyzing variation in departure probability 4. Analyzing variation in travel speed 5. Analyzing variation in total speed of migration via total stopover duration 6. Analyzing variation in total speed of migration via travel speed

### 1. General stuff

## defining work directory
wd <- "your work directory"

# set R to GMT
Sys.setenv(tz="GMT")

# Libraries
library(arm)

## Loading required package: MASS
## Loading required package: Matrix
## Loading required package: lme4
##
## arm (Version 1.8-6, built: 2015-7-7)
##
## Working directory is X:/Arbeitsgruppen/AG-Schmaljohann/Paper/NW Alaska migration/paper/spatial-specific reaction norm/Frontiers in Zoology/revision

library(blmeco)
library(geosphere)

## Loading required package: sp

library(SGAT)

## Loading required package: raster
##
## Attaching package: 'raster'
##
## The following objects are masked from 'package:MASS':
##
## area, select

library(usdm)
library(MuMIn)
library(lme4)
library(piecewiseSEM)
library(plyr)

### 2. Aggregating data

**2.a) Descriptive data about individual migration**

mig.data <- data.frame(ID.bird = c("7902", "7910", "7916", "B070", "E552", "E553", "E801", "E823"),
 lon.breed = c(-145.389167, -145.3731944, -145.3852778, -145.387000, -145.385083, -149.522676, -145.392139, -146.017583),
 lat.breed = c(65.4988056, 65.5037222, 65.4885556, 65.497028, 65.499944, 68.628406, 65.488528, 65.397083),
 onset_aut = as.POSIXct(strptime(c("2009-08-04 10:28:00", "2009-08-20 12:41:00", "2009-08-16 12:41:00", "2013-08-13 07:14:5",
 "2013-08-04 11:14:00", "2013-08-17 11:59:00", "2013-08-09 11:55:00", "2013-08-15 07:33:00"),
 format= "%Y-%m-%d %H:%M:%S"), tz="GMT"),
 onset_aut_jd = c(216, 233, 229, 226, 216, 229, 221, 227),
 term_aut = as.POSIXct(strptime(c("2009-11-14 03:11:00", "2009-11-10 15:14:00", "2009-11-12 15:11:00", "2013-11-11 03:30:00",
 "2013-11-03 03:24:00", "2013-11-18 03:23:00", "2013-11-10 13:58:00", "2013-11-27 03:30:00"),
 format= "%Y-%m-%d %H:%M:%S"), tz="GMT"),
 term_aut_jd = c(318, 315, 317, 315, 307, 322, 315, 331),
 n_stop_aut = c(10,7,10,8,8,7,5,7),
 onset_spr = as.POSIXct(strptime(c("2010-04-07 16:49:24", "2010-04-03 15:57:01", "2010-03-30 02:56:04", "2014-04-14 15:26:00",
 "2014-04-09 03:03:55", "2014-03-29 02:57:40", "2014-03-30 02:31:31", "2014-03-24 03:04:33"),
 format= "%Y-%m-%d %H:%M:%S"), tz="GMT"),
 onset_spr_jd = c(98, 94, 91, 112, 100, 89, 90, 84),
 n_stop_spr = c(3,5,5,1,2,1,4,4))

# explaining abbreviations
#-------------------------
# lon.breed: longitude of breeding area
# lat.breed: latitude of breeding area
# onset_aut: onset of autumn migration
# onset_aut_jd: onset of autumn migration in Julian date
# term_aut: termination of autumn migration
# term_aut_jd: termination of autumn migration in Julian date
# n_stop_aut: number of stopovers during autumn migration
# onset_spr: onset of spring migration
# onset_spr_jd: onset of spring migration in Julian date
# term_spr: termination of spring migration
# term_spr_jd: termination of spring migration in Julian date
# n_stop_spr: number of stopovers during spring migration

**2.b) Individual location estimates and flow assistance data**

# loading individual data, defining variables

# data are derived as detailed in
# "Appendix - R code for analyzing light-level geolocation data"

t.1 <- read.csv(paste0(wd,"7902.inddata.csv"))
t.1$time <- as.POSIXct(t.1$time)
t.1$breed <- T
t.1$breed[t.1$time >= mig.data$onset_aut[mig.data$ID.bird == "7902"]] <-F
t.1$ID.bird <- "7902"
t.1$year <- "2009"
# distance to next location as great circle distance
t.1$dist <- 0
for (i in 1:(length(t.1$time[t.1$rise==F])-1))
 t.1$dist[t.1$rise==F][i] <- round(distVincentyEllipsoid(p1 = c(t.1$Lon.mean.t[t.1$rise==F][i], t.1$Lat.mean[t.1$rise==F][i]),
 p2 = c(t.1$Lon.mean.t[t.1$rise==F][i+1], t.1$Lat.mean[t.1$rise==F][i+1]),
 a=6378137, b=6356752.3142, f=1/298.257223563)/1000,0)
for (i in 1:nrow(t.1))
 t.1$dist_breed[i] <- round(distVincentyEllipsoid(p1 = c(t.1$Lon.mean.t[i], t.1$Lat.mean[i]),
 p2 = c(mig.data$lon.breed[mig.data$ID.bird=="7902"], mig.data$lat.breed[mig.data$ID.bird=="7902"]),
 a=6378137, b=6356752.3142, f=1/298.257223563)/1000,0)

t.2 <- read.csv(paste0(wd,"7910.inddata.csv"))
t.2$time <- as.POSIXct(t.2$time)
t.2$breed <- T
t.2$breed[t.2$time >= mig.data$onset_aut[mig.data$ID.bird == "7910"]] <-F
t.2$ID.bird <- "7910"
t.2$year <- "2009"
t.2$dist <- 0
for (i in 1:(length(t.2$time[t.2$rise==F])-1))
 t.2$dist[t.2$rise==F][i] <- round(distVincentyEllipsoid(p1 = c(t.2$Lon.mean.t[t.2$rise==F][i], t.2$Lat.mean[t.2$rise==F][i]),
 p2 = c(t.2$Lon.mean.t[t.2$rise==F][i+1], t.2$Lat.mean[t.2$rise==F][i+1]),
 a=6378137, b=6356752.3142, f=1/298.257223563)/1000,0)
for (i in 1:nrow(t.2))
 t.2$dist_breed[i] <- round(distVincentyEllipsoid(p1 = c(t.2$Lon.mean.t[i], t.2$Lat.mean[i]),
 p2 = c(mig.data$lon.breed[mig.data$ID.bird=="7910"], mig.data$lat.breed[mig.data$ID.bird=="7910"]),
 a=6378137, b=6356752.3142, f=1/298.257223563)/1000,0)

t.3 <- read.csv(paste0(wd,"7916.inddata.csv"))
t.3$time <- as.POSIXct(t.3$time)
t.3$breed <- T
t.3$breed[t.3$time >= mig.data$onset_aut[mig.data$ID.bird == "7916"]] <-F
t.3$ID.bird <- "7916"
t.3$year <- "2009"
t.3$dist <- 0
for (i in 1:(length(t.3$time[t.3$rise==F])-1))
 t.3$dist[t.3$rise==F][i] <- round(distVincentyEllipsoid(p1 = c(t.3$Lon.mean.t[t.3$rise==F][i], t.3$Lat.mean[t.3$rise==F][i]),
 p2 = c(t.3$Lon.mean.t[t.3$rise==F][i+1], t.3$Lat.mean[t.3$rise==F][i+1]),
 a=6378137, b=6356752.3142, f=1/298.257223563)/1000,0)
for (i in 1:nrow(t.3))
 t.3$dist_breed[i] <- round(distVincentyEllipsoid(p1 = c(t.3$Lon.mean.t[i], t.3$Lat.mean[i]),
 p2 = c(mig.data$lon.breed[mig.data$ID.bird=="7916"], mig.data$lat.breed[mig.data$ID.bird=="7916"]),
 a=6378137, b=6356752.3142, f=1/298.257223563)/1000,0)

t.4 <- read.csv(paste0(wd,"B070.inddata.csv"))
t.4$time <- as.POSIXct(t.4$time)
t.4$breed <- T
t.4$breed[t.4$time >= mig.data$onset_aut[mig.data$ID.bird == "B070"]] <-F
t.4$ID.bird <- "B070"
t.4$year <- "2013"
t.4$dist <- 0
for (i in 1:(length(t.4$time[t.4$rise==F])-1))
 t.4$dist[t.4$rise==F][i] <- round(distVincentyEllipsoid(p1 = c(t.4$Lon.mean.t[t.4$rise==F][i], t.4$Lat.mean[t.4$rise==F][i]),
 p2 = c(t.4$Lon.mean.t[t.4$rise==F][i+1], t.4$Lat.mean[t.4$rise==F][i+1]),
 a=6378137, b=6356752.3142, f=1/298.257223563)/1000,0)
for (i in 1:nrow(t.4))
 t.4$dist_breed[i] <- round(distVincentyEllipsoid(p1 = c(t.4$Lon.mean.t[i], t.4$Lat.mean[i]),
 p2 = c(mig.data$lon.breed[mig.data$ID.bird=="B070"], mig.data$lat.breed[mig.data$ID.bird=="B070"]),
 a=6378137, b=6356752.3142, f=1/298.257223563)/1000,0)

t.5 <- read.csv(paste0(wd,"E552.inddata.csv"))
t.5$time <- as.POSIXct(t.5$time)
t.5$breed <- T
t.5$breed[t.5$time >= mig.data$onset_aut[mig.data$ID.bird == "E552"]] <-F
t.5$ID.bird <- "E552"
t.5$year <- "2013"
t.5$dist <- 0
for (i in 1:(length(t.5$time[t.5$rise==F])-1))
 t.5$dist[t.5$rise==F][i] <- round(distVincentyEllipsoid(p1 = c(t.5$Lon.mean.t[t.5$rise==F][i], t.5$Lat.mean[t.5$rise==F][i]),
 p2 = c(t.5$Lon.mean.t[t.5$rise==F][i+1], t.5$Lat.mean[t.5$rise==F][i+1]),
 a=6378137, b=6356752.3142, f=1/298.257223563)/1000,0)
for (i in 1:nrow(t.5))
 t.5$dist_breed[i] <- round(distVincentyEllipsoid(p1 = c(t.5$Lon.mean.t[i], t.5$Lat.mean[i]),
 p2 = c(mig.data$lon.breed[mig.data$ID.bird=="E552"], mig.data$lat.breed[mig.data$ID.bird=="E552"]),
 a=6378137, b=6356752.3142, f=1/298.257223563)/1000,0)

t.6 <- read.csv(paste0(wd,"E553.inddata.csv"))
t.6$time <- as.POSIXct(t.6$time)
t.6$breed <- T
t.6$breed[t.6$time >= mig.data$onset_aut[mig.data$ID.bird == "E553"]] <-F
t.6$ID.bird <- "E553"
t.6$year <- "2013"
t.6$dist <- 0
for (i in 1:(length(t.6$time[t.6$rise==F])-1))
 t.6$dist[t.6$rise==F][i] <- round(distVincentyEllipsoid(p1 = c(t.6$Lon.mean.t[t.6$rise==F][i], t.6$Lat.mean[t.6$rise==F][i]),
 p2 = c(t.6$Lon.mean.t[t.6$rise==F][i+1], t.6$Lat.mean[t.6$rise==F][i+1]),
 a=6378137, b=6356752.3142, f=1/298.257223563)/1000,0)
for (i in 1:nrow(t.6))
 t.6$dist_breed[i] <- round(distVincentyEllipsoid(p1 = c(t.6$Lon.mean.t[i], t.6$Lat.mean[i]),
 p2 = c(mig.data$lon.breed[mig.data$ID.bird=="E553"], mig.data$lat.breed[mig.data$ID.bird=="E553"]),
 a=6378137, b=6356752.3142, f=1/298.257223563)/1000,0)

t.7 <- read.csv(paste0(wd,"E801.inddata.csv"))
t.7$time <- as.POSIXct(t.7$time)
t.7$breed <- T
t.7$breed[t.7$time >= mig.data$onset_aut[mig.data$ID.bird == "E801"]] <-F
t.7$ID.bird <- "E801"
t.7$year <- "2013"
t.7$dist <- 0
for (i in 1:(length(t.7$time[t.7$rise==F])-1))
 t.7$dist[t.7$rise==F][i] <- round(distVincentyEllipsoid(p1 = c(t.7$Lon.mean.t[t.7$rise==F][i], t.7$Lat.mean[t.7$rise==F][i]),
 p2 = c(t.7$Lon.mean.t[t.7$rise==F][i+1], t.7$Lat.mean[t.7$rise==F][i+1]),
 a=6378137, b=6356752.3142, f=1/298.257223563)/1000,0)
for (i in 1:nrow(t.7))
 t.7$dist_breed[i] <- round(distVincentyEllipsoid(p1 = c(t.7$Lon.mean.t[i], t.7$Lat.mean[i]),
 p2 = c(mig.data$lon.breed[mig.data$ID.bird=="E801"], mig.data$lat.breed[mig.data$ID.bird=="E801"]),
 a=6378137, b=6356752.3142, f=1/298.257223563)/1000,0)

t.8 <- read.csv(paste0(wd,"E823.inddata.csv"))
t.8$time <- as.POSIXct(t.8$time)
t.8$breed <- T
t.8$breed[t.8$time >= mig.data$onset_aut[mig.data$ID.bird == "E823"]] <-F
t.8$ID.bird <- "E823"
t.8$year <- "2013"
t.8$dist <- 0
for (i in 1:(length(t.8$time[t.8$rise==F])-1))
 t.8$dist[t.8$rise==F][i] <- round(distVincentyEllipsoid(p1 = c(t.8$Lon.mean.t[t.8$rise==F][i], t.8$Lat.mean[t.8$rise==F][i]),
 p2 = c(t.8$Lon.mean.t[t.8$rise==F][i+1], t.8$Lat.mean[t.8$rise==F][i+1]),
 a=6378137, b=6356752.3142, f=1/298.257223563)/1000,0)
for (i in 1:nrow(t.8))
 t.8$dist_breed[i] <- round(distVincentyEllipsoid(p1 = c(t.8$Lon.mean.t[i], t.8$Lat.mean[i]),
 p2 = c(mig.data$lon.breed[mig.data$ID.bird=="E823"], mig.data$lat.breed[mig.data$ID.bird=="E823"]),
 a=6378137, b=6356752.3142, f=1/298.257223563)/1000,0)

**2.c) Individual weather data**

#
# Look at AK wheater ind environ data.rmd file
#
#
# loading individual weather data

t.9a <- read.csv(paste0(wd,"7902.weather.data.csv"))
t.10a <- read.csv(paste0(wd,"7910.weather.data.csv"))
t.11a <- read.csv(paste0(wd,"7916.weather.data.csv"))
t.12a <- read.csv(paste0(wd,"B070.weather.data.csv"))
t.13a <- read.csv(paste0(wd,"E552.weather.data.csv"))
t.14a <- read.csv(paste0(wd,"E553.weather.data.csv"))
t.15a <- read.csv(paste0(wd,"E801.weather.data.csv"))
t.16a <- read.csv(paste0(wd,"E823.weather.data.csv"))

# loading individual surface wind speed data

t.9b <- read.csv(paste0(wd,"7902.indwinddata.csv"))
t.10b <- read.csv(paste0(wd,"7910.indwinddata.csv"))
t.11b <- read.csv(paste0(wd,"7916.indwinddata.csv"))
t.12b <- read.csv(paste0(wd,"B070.indwinddata.csv"))
t.13b <- read.csv(paste0(wd,"E552.indwinddata.csv"))
t.14b <- read.csv(paste0(wd,"E553.indwinddata.csv"))
t.15b <- read.csv(paste0(wd,"E801.indwinddata.csv"))
t.16b <- read.csv(paste0(wd,"E823.indwinddata.csv"))

t.9 <- cbind(t.9a, t.9b)
t.10 <- cbind(t.10a, t.10b)
t.11 <- cbind(t.11a, t.11b)
t.12 <- cbind(t.12a, t.12b)
t.13 <- cbind(t.13a, t.13b)
t.14 <- cbind(t.14a, t.14b)
t.15 <- cbind(t.15a, t.15b)
t.16 <- cbind(t.16a, t.16b)

**2.d) Combining location estimates, flow assistance data, and weather data**

a.data <- rbind(cbind(t.1,t.9),cbind(t.2,t.10),cbind(t.3,t.11),cbind(t.4,t.12),cbind(t.5,t.13),cbind(t.6,t.14),cbind(t.7,t.15),cbind(t.8,t.16))

# ordering number
a.data$order <- 1:nrow(a.data)

# adjusting data and calculating new variables
 a.data$time <- as.POSIXct(a.data$time)
 a.data$ID.bird <- as.factor(a.data$ID.bird)
 # Julian date
 a.data$jd <- strptime(a.data$time, "%Y-%m-%d %H:%M:%S")$yday+1
 # Season
 a.data$season <- as.factor(ifelse(a.data$jd<180,"spr","aut"))
 # Year
 a.data$year <- as.factor(a.data$year)
 # Distance to migratory goal
 for (b in 1:8)
 {
 t.sel <- c("7902","7910","7916","B070","E552","E553","E801","E823")[b]
 a.data$dist_mig_goal[a.data$ID.bird == t.sel] <- ifelse(a.data[a.data$ID.bird == t.sel,"season"] == "aut",
 max(a.data[a.data$ID.bird == t.sel,"dist_breed"]) - a.data[a.data$ID.bird == t.sel,"dist_breed"],
 a.data[a.data$ID.bird == t.sel,"dist_breed"])
 }

### 3. Analyzing variation in departure probability

**3.a) Preparing data for both seasons**

# number of days during migration, i.e., excluding times at both the breeding area and wintering ground:
length(which(!is.na(a.data$fa.nmigg[a.data$breed == F & a.data$winter == F & a.data$rise == F])))

## [1] 1038

# number of days during migration on which flow assistance
# could NOT be estimated:
 # Before estimating flow assistance the variable "fa.nmigg"
 # was set arbitrarily to 9999.
 # The number of cases in which "fa.nmigg" has the value of 9999
 # gives the number of nights on which flow assistance could
 # not be estimated.
 length(which(a.data$fa.nmigg==9999))

## [1] 48

# a.data$fa.nmigg <- ifelse(a.data$fa.nmigg==9999,NA,a.data$fa.nmigg)

# departure probability data
# As we estimated the flow assistance in accordance with the "NCEP.Airspeed" concept (Kemp et al. 2012a, b) by the "NCEP.flight"" function, flow assistance could not be estimated when wind speed exceeded the assumed airspeed of 13 m/s in northern wheatears, following Bruderer & Boldt (2001).
 # times when birds were either at the breeding area or at the wintering grounds had to be excluded
 # only sunset events were considered
 dp.data <- a.data[a.data$winter==F & a.data$breed==F & a.data$rise==F &
 a.data$fa.nmigg!=9999 &
 a.data$wnd.speed.surface!=9999,]

# omitting the 48 "bird-dates" on which flow assistance could not be estimated
 # check
 length(which(dp.data$fa.nmigg==9999))

## [1] 0

# defining on which night birds set off for a migratory flight
 # migratory state "stopping" is set to "0"
 # migratory state "migrating" is set to "1"
 dp.data$dep.prob <- as.integer(ifelse(dp.data$state=="stopping",0,1))

# Z-transformation of variables
dp.data$air.temp.s <- scale(dp.data$air.temp)
dp.data$rain.s <- scale(dp.data$rain)
dp.data$air.press.s <- scale(dp.data$air.press)
dp.data$wnd.speed.surface.s <- scale(dp.data$wnd.speed.surface)
dp.data$fa.nmigg.s <- scale(dp.data$fa.nmigg)
dp.data$dist_mig_goal.s <- scale(dp.data$dist_mig_goal)

**3.b) Selecting autumn data**

a.dp.data <- dp.data[dp.data$season == "aut",]

**3.c) Analyzing autumn data**

Assessing collinearity Parameters with values > 2 will be removed, cf. Zuur et al. (2010). As we are mainly interested in whether birds' reaction to environmental cues changed with the remaining migration distance, the variable(s) that is (are) collinear with the remaining migration distance is (are) removed. This means that the potential effect of the remaining migration distance on the variation in travel speed could not be separated from an effect of the collinear variables.

vif(a.dp.data[,c("air.temp.s", "rain.s", "air.press.s", "wnd.speed.surface.s", "fa.nmigg.s", "dist_mig_goal.s" )])

## Variables VIF
## 1 air.temp.s 1.475677
## 2 rain.s 1.046614
## 3 air.press.s 1.184798
## 4 wnd.speed.surface.s 1.050624
## 5 fa.nmigg.s 1.040384
## 6 dist_mig_goal.s 1.575068

# --> good, all values below 2

# Visualizing explanatory variables
pairs(a.dp.data[,c("air.temp.s", "rain.s", "air.press.s", "wnd.speed.surface.s", "fa.nmigg.s", "dist_mig_goal.s" )])


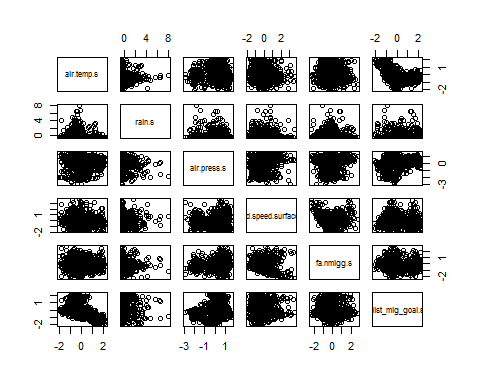


# Running model
a.dp.mod <- glmer(dep.prob ~ air.temp.s*dist_mig_goal.s +
 rain.s*dist_mig_goal.s +
 air.press.s*dist_mig_goal.s +
 wnd.speed.surface.s*dist_mig_goal.s +
 fa.nmigg.s*dist_mig_goal.s +
 (1|year),
 data=a.dp.data,
 family="binomial",
 na.action=na.omit)

# Sample size
length(a.dp.mod@resp$n)

## [1] 694

**3.d) Assessing model assumptions: autumn**

# Checking for overdispersion
dispersion_glmer(a.dp.mod)

## [1] 1.059767

The scale parameter is between 0.75 and 1.4. There is not indication of overdispersion, cf. Nievergelt et al. (2015).

# residual analysis
par(mfrow=c(2,2))
scatter.smooth(fitted(a.dp.mod), resid(a.dp.mod)); abline(h=0, lty=2)
mtext("Tukey-Anscombe Plot", 3, line=0.8, cex=0.8)
qqnorm(resid(a.dp.mod), main="Normal QQ plot, residuals", cex.main=0.8)
qqline(resid(a.dp.mod))
scatter.smooth(fitted(a.dp.mod), sqrt(abs(resid(a.dp.mod))))
qqnorm(ranef(a.dp.mod)$year[,1], main="Normal QQ plot, random effect, years", cex.main=0.8)
qqline(ranef(a.dp.mod)$year[,1])


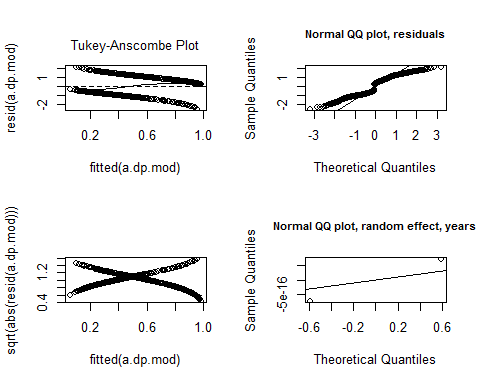


Diagnostic residual plots to assess model assumptions. Upper left: Residuals over predicted values. Upper right: Normal QQ plot of the residuals. Lower left: Square-root of the absolute values of the residuals versus fitted values. Lower left: Normal QQ plot of the random effect, year. Due to the binary nature of the response variable (here departure probability) the residual plots usually look a bit strange of mixed effect logistic regression models. In the Tukey-Anscombe plot residuals average roughly around zero. Both QQ plots do not show serious deviation from model's assumption. In all, the residual analysis looks acceptable.

# According to Nievergelt et al. (2015) the function "glmer" sometimes produces wrong results
# without giving a warning. This can be assessed by estimating the mean of the random effect.
mean(ranef(a.dp.mod)$year[,1])

## [1] 5.113493e-22

# That values seems acceptable, because it will be added to the overall mean. Thus, the estimate
# for the overall mean will be 0.0003 too low.
# This translates to an underestimation of the overall mean departure probability of about
# 0.02%. This is negligible here.
t.should <- plogis(fixef(a.dp.mod)["(Intercept)"])
t.is <- plogis(fixef(a.dp.mod)["(Intercept)"] - mean(ranef(a.dp.mod)$year[,1]))
# => the error at the Intercept is:
(t.should-t.is)/t.should

## (Intercept)
## 0

# or:
(1-exp(mean(ranef(a.dp.mod)$year[,1])))

## [1] 0

# the multiplicative factor on the odds (i.e. departure/stopover) is about 0%

# goodness of fit plot
plot(fitted(a.dp.mod)[!is.na(a.dp.data$dep.prob)],
 jitter(a.dp.data$dep.prob[!is.na(a.dp.data$dep.prob)], amount=0.05),
 xlab="Fitted values", ylab="Probability of presence",
 xlim=c(0,1),
 las=1, cex.lab=1.2, cex=0.8)
abline(0,1, lty=3)
t.breaks <- cut(fitted(a.dp.mod)[!is.na(a.dp.data$dep.prob)], seq(0,1, by=0.2))
means <- tapply(a.dp.data$dep.prob[!is.na(a.dp.data$dep.prob)], t.breaks, mean)
semean <- function(x) sd(x)/sqrt(length(x))
means.se <- tapply(a.dp.data$dep.prob[!is.na(a.dp.data$dep.prob)], t.breaks, semean)
points(seq(0.05, 0.95, by=0.2), means, pch=16, col="orange")
segments(seq(0.05, 0.95, by=0.2), means-2*means.se, seq(0.05, 0.95, by=0.2), means+2*means.se,lwd=2, col="orange")


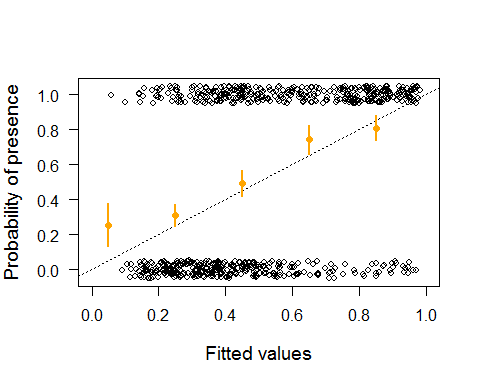


Goodness of fit plot. Probability of stopover versus fitted values. Open circles = observed stopover (0) or departure (1) jittere in the vertical direction; organe dots = mean (and 95% confidence intervals given as vertical bars) of the observations within classes of width 0.1 along the x-axis. The dotted line indicates perfect coincidence between observations and fitted values (Nievergelt et al. 2015). This is a good fit.

**3.e) Drawing conclusions form the generalized lineas mixed model: autumn**

# Estimates and 95% CrI of the parameters using sim
nsim <- 2000
set.seed(111) # seed was specified for reproducibility
bsim <- sim(a.dp.mod, n.sim=nsim)
round(apply(bsim@fixef,2,quantile,prob=c(0.025,0.5,0.975)),2)

## (Intercept) air.temp.s dist_mig_goal.s rain.s air.press.s
## 2.5% -0.46 -0.56 0.73 -0.05 -0.32
## 50% -0.25 -0.30 0.98 0.14 -0.09
## 97.5% -0.04 -0.04 1.23 0.32 0.13
## wnd.speed.surface.s fa.nmigg.s air.temp.s:dist_mig_goal.s
## 2.5% -0.17 -0.53 -1.11
## 50% 0.00 -0.33 -0.83
## 97.5% 0.19 -0.12 -0.57
## dist_mig_goal.s:rain.s dist_mig_goal.s:air.press.s
## 2.5% -0.13 -0.57
## 50% 0.07 -0.30
## 97.5% 0.25 -0.01
## dist_mig_goal.s:wnd.speed.surface.s dist_mig_goal.s:fa.nmigg.s
## 2.5% -0.01 -0.20
## 50% 0.20 0.06
## 97.5% 0.41 0.31

These values are displayed in Tab. 2.

**3.f) Selecting spring data: spring**

s.dp.data <- dp.data[dp.data$season == "spr",]

**3.g) Analyzing spring data**

Assessing collinearity Parameters with values > 2 will be removed, cf. Zuur et al. (2010). As we are mainly interested in whether birds' reaction to environmental cues changed with the remaining migration distance, the variable(s) that is (are) collinear with the remaining migration distance is (are) removed. This means that the potential effect of the remaining migration distance on the variation in travel speed could not be separated from an effect of the collinear variables.

vif(s.dp.data[,c("air.temp.s", "rain.s", "air.press.s", "wnd.speed.surface.s", "fa.nmigg.s", "dist_mig_goal.s" )])

## Variables VIF
## 1 air.temp.s 3.787159
## 2 rain.s 1.093157
## 3 air.press.s 1.230768
## 4 wnd.speed.surface.s 1.073928
## 5 fa.nmigg.s 1.052114
## 6 dist_mig_goal.s 4.018018

# --> drop air.temp.s

vif(s.dp.data[,c("rain.s", "air.press.s", "wnd.speed.surface.s", "fa.nmigg.s", "dist_mig_goal.s" )])

## Variables VIF
## 1 rain.s 1.053061
## 2 air.press.s 1.116712
## 3 wnd.speed.surface.s 1.046475
## 4 fa.nmigg.s 1.052057
## 5 dist_mig_goal.s 1.121679

# --> good, all values below 2

# Visualizing explanatory variables
pairs(s.dp.data[,c( "rain.s", "air.press.s", "wnd.speed.surface.s", "fa.nmigg.s", "dist_mig_goal.s" )])


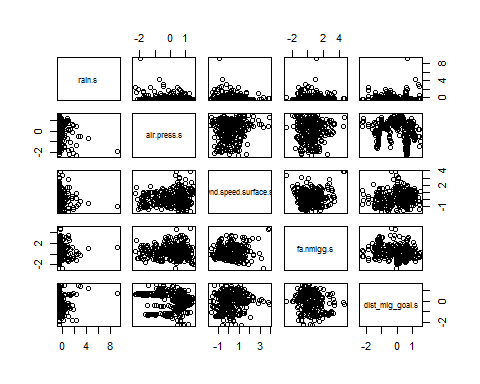


# Running model
s.dp.mod <- glmer(dep.prob ~ rain.s*dist_mig_goal.s +
 air.press.s*dist_mig_goal.s +
 fa.nmigg.s*dist_mig_goal.s +
 wnd.speed.surface.s*dist_mig_goal.s +
 (1|year),
 data=s.dp.data,
 family="binomial")

# Sample size
length(s.dp.mod@resp$n)

## [1] 297

**3.h) Assessing model assumptions: spring**

# Checking for overdispersion
dispersion_glmer(s.dp.mod)

## [1] 0.9765928

The scale parameter is between 0.75 and 1.4. There is not indication of overdispersion, cf. Nievergelt et al. (2015).

# residual analysis
par(mfrow=c(2,2))
scatter.smooth(fitted(s.dp.mod), resid(s.dp.mod)); abline(h=0, lty=2)
mtext("Tukey-Anscombe Plot", 3, line=0.8, cex=0.8)
qqnorm(resid(s.dp.mod), main="Normal QQ plot, residuals", cex.main=0.8)
qqline(resid(s.dp.mod))
scatter.smooth(fitted(s.dp.mod), sqrt(abs(resid(s.dp.mod))))
qqnorm(ranef(s.dp.mod)$year[,1], main="Normal QQ plot, random effect, years", cex.main=0.8)
qqline(ranef(s.dp.mod)$year[,1])


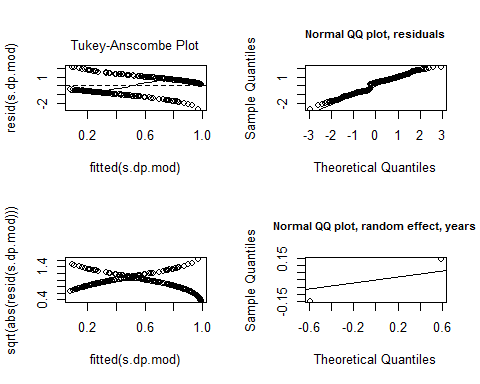


Diagnostic residual plots to assess model assumptions. Upper left: Residuals over predicted values. Upper right: Normal QQ plot of the residuals. Middle left: Square-root of the absolute values of the residuals versus fitted values. Middle right: Normal QQ plot of the random effects, birds nested in years. Lower left: Normal QQ plot of the random effect, years. Due to the binary nature of the response variable (here departure probability) the residual plots usually look a bit strange of mixed effect logistic regression models. In the Tukey-Anscombe plot residuals average roughly around zero. Both QQ plots do not show serious deviation from model's assumption. In all, the residual analysis looks acceptable.

# According to Nievergelt et al. (2015) the function "glmer" sometimes produces wrong results
# without giving a warning. This can be assessed by estimating the mean of the random effect.
mean(ranef(s.dp.mod)$year[,1])

## [1] -0.0002867424

# That values seems acceptable, because it will be added to the overall mean. Thus, the estimate
# for the overall mean will be 0.004 too high.
# This translates to an underesimation of the overall mean departure probability of about
# 0.11%. This is negligible here.
t.should <- plogis(fixef(s.dp.mod)["(Intercept)"])
t.is <- plogis(fixef(s.dp.mod)["(Intercept)"] - mean(ranef(s.dp.mod)$year[,1]))
# => the error at the Intercept is:
(t.should-t.is)/t.should

## (Intercept)
## -0.0001156823

# or:
(1-exp(mean(ranef(s.dp.mod)$year[,1])))

## [1] 0.0002867013

# the multiplicative factor on the odds (i.e. departure/stopover) is about 0.4% too large

# goodness of fit plot
plot(fitted(s.dp.mod)[!is.na(s.dp.data$dep.prob)],
 jitter(s.dp.data$dep.prob[!is.na(s.dp.data$dep.prob)], amount=0.05),
 xlab="Fitted values", ylab="Probability of stopover", las=1, cex.lab=1.2, cex=0.8,
 xlim=c(0,1))
abline(0,1, lty=3)
t.breaks <- cut(fitted(s.dp.mod)[!is.na(s.dp.data$dep.prob)], seq(0,1, by=0.2))
means <- tapply(s.dp.data$dep.prob[!is.na(s.dp.data$dep.prob)], t.breaks, mean)
semean <- function(x) sd(x)/sqrt(length(x))
means.se <- tapply(s.dp.data$dep.prob[!is.na(s.dp.data$dep.prob)], t.breaks, semean)
points(seq(0.05, 0.95, by=0.2), means, pch=16, col="orange")
segments(seq(0.05, 0.95, by=0.2), means-2*means.se, seq(0.05, 0.95, by=0.2), means+2*means.se,lwd=2, col="orange")


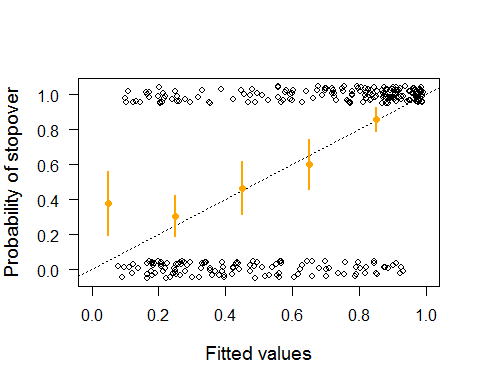


Goodness of fit plot. Probability of stopover versus fitted values. Open circles = observed stopover (0) or departure (1) jittere in the vertical direction; organe dots = mean (and 95% confidence intervals given as vertical bars) of the observations within classes of width 0.2 along the x-axis.The dotted line indicates perfect coincidence between observations and fitted values (Nievergelt et al. 2015).

**3.i) Drawing conclusions form the generalized lineas mixed model: spring**

# Estimates and 95% CrI of the parameters using sim
nsim <- 2000
set.seed(111) # seed was specified for reproducibility
bsim <- sim(s.dp.mod, n.sim=nsim)
colnames(bsim@fixef) <- names(fixef(s.dp.mod))
round(apply(bsim@fixef,2,quantile,prob=c(0.025,0.5,0.975)),2)

## (Intercept) rain.s dist_mig_goal.s air.press.s fa.nmigg.s
## 2.5% -0.05 -0.47 -1.81 0.34 0.14
## 50% 0.39 0.02 -1.34 0.70 0.42
## 97.5% 0.82 0.46 -0.86 1.07 0.72
## wnd.speed.surface.s rain.s:dist_mig_goal.s
## 2.5% -0.61 -0.48
## 50% -0.30 0.06
## 97.5% 0.02 0.57
## dist_mig_goal.s:air.press.s dist_mig_goal.s:fa.nmigg.s
## 2.5% -0.50 -0.70
## 50% 0.03 -0.23
## 97.5% 0.60 0.24
## dist_mig_goal.s:wnd.speed.surface.s
## 2.5% -0.52
## 50% 0.04
## 97.5% 0.57

These values are displayed in Tab. 2.

### 4. Analyzing variation in travel speed

**4.**

#selecting data for travel speed analses
# selecting for each individual migration data by omitting location estimates
# from both the breeding areas and wintering grounds and also from stopovers

travel.data <- a.data[a.data$winter == F & a.data$breed == F &
 a.data$rise == F & a.data$state=="migrating",]

# z-transformation of variables
# explaing abbreviation
#----------------------
# airsp.fa:
# This is the hourly mean of the flow assistance experienced by the birds
# to reach next location estimate the next day.
# For details see 5. Analyses of phenotypic response to environmental cues along the migration route
# in the supplemental materials.

travel.data$air.temp.s <- scale(travel.data$air.temp)
travel.data$rain.s <- scale(travel.data$rain)
travel.data$air.press.s <- scale(travel.data$air.press)
travel.data$airsp.fa.s <- scale(travel.data$airsp.fa)
travel.data$dist_mig_goal.s <- scale(travel.data$dist_mig_goal)

**4.a) Selecting autumn data**

# autumn
aut.data <- travel.data[travel.data$season=="aut",]

**4.b) Analyzing autumn data**

Assessing collinearity Parameters with values > 2 will be removed, cf. Zuur et al. (2010). As we are mainly interested in whether birds' reaction to environmental cues changed with the remaining migration distance, the variable(s) that is (are) collinear with the remaining migration distance is (are) removed. This means that the potential effect of the remaining migration distance on the variation in travel speed could not be separated from an effect of the collinear variables.

vif(aut.data[,c("air.temp.s","rain.s","air.press.s","airsp.fa.s","dist_mig_goal.s")] )

## Variables VIF
## 1 air.temp.s 1.571617
## 2 rain.s 1.037599
## 3 air.press.s 1.088477
## 4 airsp.fa.s 1.021214
## 5 dist_mig_goal.s 1.650753

# --> good, all values below 2

# visualizing explanatory variables
pairs(aut.data[,c("air.temp.s","rain.s","air.press.s","airsp.fa.s","dist_mig_goal.s")] )


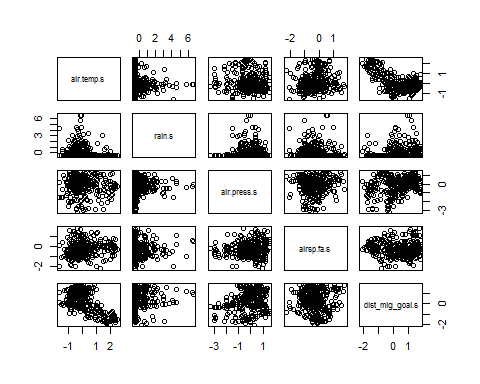


# linear mixed effect model for autumn
######################################
# The variable "dist" gives the information of the individual distance
# travelled per night.
# Here it was log10 transformed so that
# model assumptions were not violated.

mod.at <- lmer(log10(dist) ~ air.temp.s*dist_mig_goal.s +
 rain.s*dist_mig_goal.s +
 air.press.s*dist_mig_goal.s +
 airsp.fa.s*dist_mig_goal.s +
 (1|year),
 data = aut.data,
 weight = 1/aut.data$Lat.sd)

# sample size
length(mod.at@resp$mu)

## [1] 303

**4.c) Assessing model assumptions: autumn**

# residual analysis
par(mfrow=c(2,2))
scatter.smooth(fitted(mod.at), resid(mod.at)); abline(h=0, lty=2)
mtext("Tukey-Anscombe Plot", 3, line=0.8, cex=0.8)
qqnorm(resid(mod.at), main="Normal QQ-plot, residuals", cex.main=0.9)
qqline(resid(mod.at))
scatter.smooth(fitted(mod.at), sqrt(abs(resid(mod.at)))); abline(h=0, lty=2)
qqnorm(ranef(mod.at)$year[,1], main="Normal QQ plot, random effect, years", cex.main=0.8)
qqline(ranef(mod.at)$year[,1])


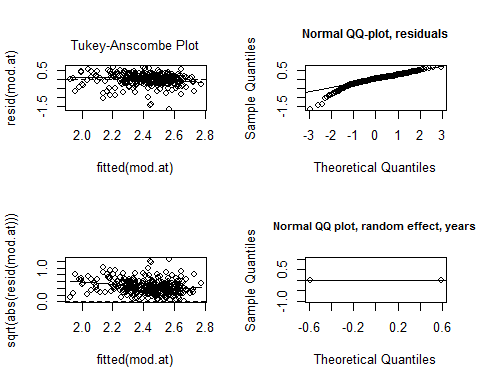


Diagonstic residual plots to assess model assumptions. Upper left: Residuals over predicted values. Upper right: Normal QQ plot of the residuals. Lower left: Square-root of the absolute values of the residuals versus fitted values. Lower right: Normal QQ plot of the random effects, years. In the Tukey-Anscombe plot there is a slight decrease of the residuals with the fitted values. The QQ plot of the residuals show a banana-type of curve. Nevertheless, the plots show no serious deviation from model assumptions in our opinion.

**4.d) Drawing conclusions from the model: autumn**

# estimates and 95% CrI of the parameters using sim
nsim <- 2000
set.seed(0470) # seed was specified for reproducibility
bsim <- sim(mod.at, n.sim=nsim)
colnames(bsim@fixef) <- names(fixef(mod.at))
# from the simulated values, the 2.5% and 97.5% quantiles can be used for the 95% credible interval
round(apply(bsim@fixef, 2, quantile, prob=c(0.025,0.5,0.975)),2)

## (Intercept) air.temp.s dist_mig_goal.s rain.s air.press.s airsp.fa.s
## 2.5% 2.37 -0.18 -0.22 -0.06 0.03 -0.12
## 50% 2.45 -0.12 -0.16 -0.01 0.08 -0.04
## 97.5% 2.52 -0.07 -0.10 0.05 0.12 0.04
## air.temp.s:dist_mig_goal.s dist_mig_goal.s:rain.s
## 2.5% -0.11 -0.05
## 50% -0.06 0.00
## 97.5% -0.01 0.05
## dist_mig_goal.s:air.press.s dist_mig_goal.s:airsp.fa.s
## 2.5% -0.01 -0.10
## 50% 0.03 -0.03
## 97.5% 0.07 0.03

These values are displayed in Tab. 3.

**4.e) Selecting spring data**

spr.data <- travel.data[travel.data$season=="spr",]

**4.f) Analyzing spring data**

Assessing collinearity Parameters with values > 2 will be removed, cf. Zuur et al. (2010). As we are mainly interested in whether birds' reaction to environmental cues changed with the remaining migration distance, the variable(s) that is (are) collinear with the remaining migration distance is (are) removed. This means that the potential effect of the remaining migration distance on the variation in travel speed could not be separated from an effect of the collinear variables.

vif(spr.data[,c("air.temp.s","rain.s","air.press.s","airsp.fa.s","dist_mig_goal.s")])

## Variables VIF
## 1 air.temp.s 3.465379
## 2 rain.s 1.074268
## 3 air.press.s 1.101583
## 4 airsp.fa.s 1.038968
## 5 dist_mig_goal.s 3.317215

# --> drop air temperature

vif(spr.data[,c("rain.s","air.press.s","airsp.fa.s","dist_mig_goal.s")])

## Variables VIF
## 1 rain.s 1.067964
## 2 air.press.s 1.029291
## 3 airsp.fa.s 1.038394
## 4 dist_mig_goal.s 1.105213

# --> good, all values below 2

# visualizing explanatory variables
pairs(spr.data[,c("rain.s","air.press.s","airsp.fa.s","dist_mig_goal.s")])


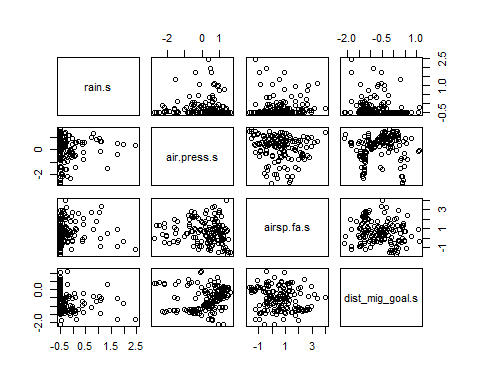


# linear mixed effect model for spring
######################################
mod.sp <- lmer(log10(dist) ~ rain.s*dist_mig_goal.s +
 air.press.s*dist_mig_goal.s +
 airsp.fa.s*dist_mig_goal.s +
 (1|year),
 data = spr.data,
 weight = 1/spr.data$Lat.sd)

# sample size
length(mod.sp@resp$mu)

## [1] 153

**4.g) Assessing model assumptions: spring**

# residual analysis
par(mfrow=c(2,2))
scatter.smooth(fitted(mod.sp), resid(mod.sp)); abline(h=0, lty=2)
mtext("Tukey-Anscombe Plot", 3, line=0.8, cex=0.8)
qqnorm(resid(mod.sp), main="Normal QQ-plot, residuals", cex.main=0.9)
qqline(resid(mod.sp))
scatter.smooth(fitted(mod.sp), sqrt(abs(resid(mod.sp)))); abline(h=0, lty=2)
qqnorm(ranef(mod.sp)$year[,1], main="Normal QQ plot, random effect, years", cex.main=0.8)
qqline(ranef(mod.sp)$year[,1])


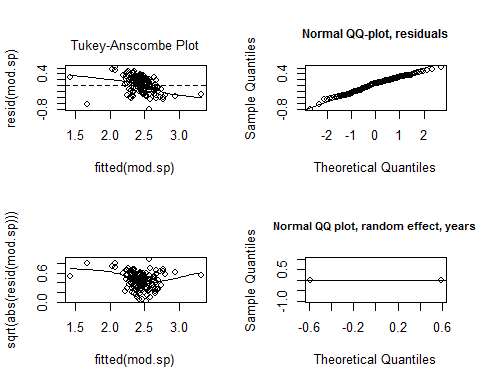


Diagonstic residual plots to assess model assumptions. Upper left: Residuals over predicted values. Upper right: Normal QQ plot of the residuals. Lower left: Square-root of the absolute values of the residuals versus fitted values. Lower left: Normal QQ plot of the random effects, years. In the Tukey-Anscombe plot we see that the residuals generally scatter around zero, but also that the residuals are slightly negatively correlated with the fitted values. As the other plots show no deviation from model assumptions (Nievergelt et al. 2015), we accepted this model.

**4.h) Drawing conclusions from the model: spring**

# estimates and 95% CrI of the parameters using sim
nsim <- 2000
set.seed(0470) # seed was specified for reproducibility
bsim <- sim(mod.sp, n.sim=nsim)
colnames(bsim@fixef) <- names(fixef(mod.sp))
# from the simulated values, the 2.5% and 97.5% quantiles can be used for the 95% credible interval
round(apply(bsim@fixef, 2, quantile, prob=c(0.025,0.5,0.975)),3)

## (Intercept) rain.s dist_mig_goal.s air.press.s airsp.fa.s
## 2.5% 2.544 0.116 0.160 -0.106 -0.227
## 50% 2.690 0.359 0.265 -0.009 -0.132
## 97.5% 2.827 0.591 0.371 0.092 -0.042
## rain.s:dist_mig_goal.s dist_mig_goal.s:air.press.s
## 2.5% 0.186 -0.135
## 50% 0.349 -0.056
## 97.5% 0.503 0.024
## dist_mig_goal.s:airsp.fa.s
## 2.5% -0.217
## 50% -0.143
## 97.5% -0.074

These values are displayed in Tab. 3.

### 5. Analyzing variation in total speed of migration via total stopover duration

speed.data <- read.csv(paste0(wd,"totalspeed stopover_dur travel_speed.csv"))

**5.a) Seasonal differences in tracked migration distances**

# migration distance tracked
 # autumn
 round(mean(speed.data$w.sp[speed.data$season=="aut"]),0)

## [1] 14789

round(sd(speed.data$w.sp[speed.data$season=="aut"]),0)

## [1] 1757

# spring
 round(mean(speed.data$w.sp[speed.data$season=="spr"]),0)

## [1] 7928

round(sd(speed.data$w.sp[speed.data$season=="spr"]),0)

## [1] 1865

**5.b) Analyzing data**

# linear mixed effect model
###########################
mod0.ts <- lmer(log10(t.sp.m) ~ log10(sp)+season+(1|ID),speed.data, REML=T)

# sample size
length(mod0.ts@resp$mu)

## [1] 16

**5.c) Assessing model assumptions**

# residual analysis
par(mfrow=c(2,2), mar=c(4,4,2,1), mgp=c(2.2,0.8,0))
scatter.smooth(fitted(mod0.ts), resid(mod0.ts)); abline(h=0, lty=2)
mtext("Tukey-Anscombe Plot", 3, line=0.8, cex=0.8)
qqnorm(resid(mod0.ts), main="normal qq-plot, residuals", cex.main=0.8)
qqline(resid(mod0.ts))
scatter.smooth(fitted(mod0.ts), sqrt(abs(resid(mod0.ts))))
qqnorm(ranef(mod0.ts)$ID[,1], main="normal qq-plot, random effects", cex.main=0.8)
qqline(ranef(mod0.ts)$ID[,1])


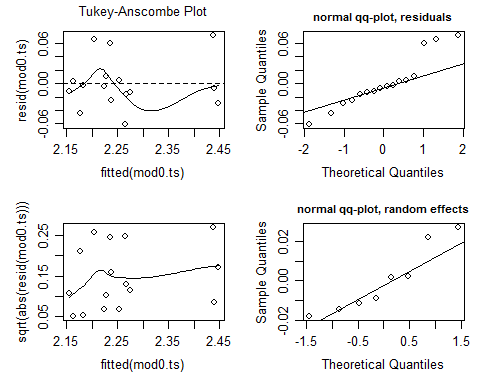


Diagonstic residual plots to assess model assumptions. upper left: Residuals over predicted values. Upper right: Normal QQ plot of the residuals. Lower left: Square-root of the absolute values of the residuals versus fitted values. Lower right: Normal QQ plot of the random effect, birds In the Tukey-Anscombe plot we see that the residuals generally scatter around zero. In the QQ plots several data points do not fit perfectly well to the QQ line. As the other plots show no serious deviation from model assumptions (Nievergelt et al. 2015), we still accepted this model.

**5.d) Drawing conclusions from model:**

#draw 2000 random values from the joint posterior distribution to obtain CrI
set.seed(0470) # specify the seed (starting value for your random generator)
nsim <- 2000
bsim <- sim(mod0.ts, n.sim=nsim)
colnames(bsim@fixef) <- names(fixef(mod0.ts)) # sim does not assign the names of the parameter in the current version.

#From the simulated values, the 2.5% and 97.5% quantiles can be used for the 95% credible interval
round(apply(bsim@fixef, 2, quantile, prob=c(0.025,0.5,0.975)),2)

## (Intercept) log10(sp) seasonspr
## 2.5% 2.63 -0.59 -0.17
## 50% 2.89 -0.42 -0.08
## 97.5% 3.17 -0.27 0.01

# Note that the effect of season on total speed of migration was not significant.
# We therefore simplify the model here.

**5.e) Simplyfing the model:**

# linear mixed effect model
###########################
mod1.ts <- lmer(log10(t.sp.m) ~ log10(sp)+(1|ID),speed.data, REML=T)

# comparing models
anova(mod0.ts, mod1.ts)

## refitting model(s) with ML (instead of REML)

## Data: speed.data
## Models:
## mod1.ts: log10(t.sp.m) ~ log10(sp) + (1 | ID)
## mod0.ts: log10(t.sp.m) ~ log10(sp) + season + (1 | ID)
## Df AIC BIC logLik deviance Chisq Chi Df Pr(>Chisq)
## mod1.ts 4 -41.311 -38.221 24.656 -49.311
## mod0.ts 5 -42.571 -38.708 26.285 -52.571 3.2595 1 0.07101 .
## ---
## Signif. codes: 0 '***' 0.001 '**' 0.01 '*' 0.05 '.' 0.1 ' ' 1

# As the more complex model was not significantly better than the more simple model
# and as season was not significant, we work from now on with mod1.

**5.f) Assessing model assumptions**

# residual analysis
par(mfrow=c(2,2), mar=c(4,4,2,1), mgp=c(2.2,0.8,0))
scatter.smooth(fitted(mod1.ts), resid(mod1.ts)); abline(h=0, lty=2)
mtext("Tukey-Anscombe Plot", 3, line=0.8, cex=0.8)
qqnorm(resid(mod1.ts), main="normal qq-plot, residuals", cex.main=0.8)
qqline(resid(mod1.ts))
scatter.smooth(fitted(mod1.ts), sqrt(abs(resid(mod1.ts))))
qqnorm(ranef(mod1.ts)$ID[,1], main="normal qq-plot, random effects", cex.main=0.8)
qqline(ranef(mod1.ts)$ID[,1])


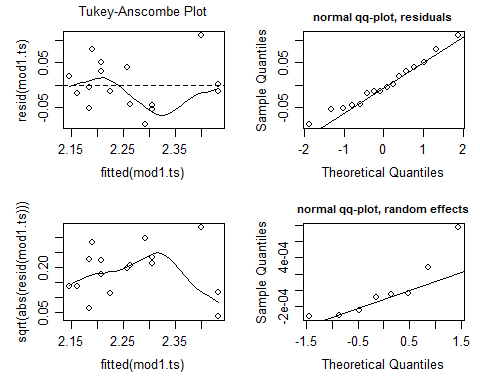


Diagonstic residual plots to assess model assumptions. upper left: Residuals over predicted values. Upper right: Normal QQ plot of the residuals. Lower left: Square-root of the absolute values of the residuals versus fitted values. Lower right: Normal QQ plot of the random effect, birds In the Tukey-Anscombe plot we see that the residuals generally scatter around zero. In the normal QQ plot of the random effect two data points do not fit perfectly well to the QQ line. As the other plots show no serious deviation from model assumptions (Nievergelt et al. 2015), we accepted this model.

**5.g) Drawing conclusions from model:**

#draw 2000 random values from the joint posterior distribution to obtain CrI
set.seed(0470) # specify the seed (starting value for your random generator)
nsim <- 2000
bsim.ts <- sim(mod1.ts, n.sim=nsim)
colnames(bsim.ts@fixef) <- names(fixef(mod1.ts)) # sim does not assign the names of the parameter in the current version.

#From the simulated values, the 2.5% and 97.5% quantiles can be used for the 95% credible interval
round(apply(bsim.ts@fixef, 2, quantile, prob=c(0.025,0.5,0.975)),2)

## (Intercept) log10(sp)
## 2.5% 2.54 -0.41
## 50% 2.69 -0.31
## 97.5% 2.84 -0.20

### 6. Analyzing variation in total speed of migration via mean travel speed

**6.a) Analyzing data**

# linear mixed effect model
###########################
mod0.sp <- lmer(log10(t.sp.m) ~ log10(travel.sp)+season+(1|ID),speed.data, REML=T)

# sample size
length(mod0.sp@resp$mu)

## [1] 16

**6.b) Assessing model assumptions**

# residual analysis
par(mfrow=c(2,2), mar=c(4,4,2,1), mgp=c(2.2,0.8,0))
scatter.smooth(fitted(mod0.sp), resid(mod0.sp)); abline(h=0, lty=2)
mtext("Tukey-Anscombe Plot", 3, line=0.8, cex=0.8)
qqnorm(resid(mod0.sp), main="normal qq-plot, residuals", cex.main=0.8)
qqline(resid(mod0.sp))
scatter.smooth(fitted(mod0.sp), sqrt(abs(resid(mod0.sp))))
qqnorm(ranef(mod0.sp)$ID[,1], main="normal qq-plot, random effects", cex.main=0.8)
qqline(ranef(mod0.sp)$ID[,1])


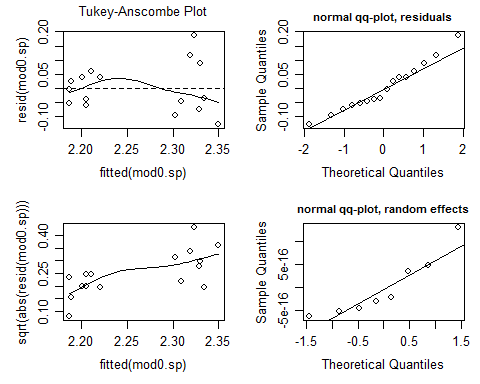


Diagonstic residual plots to assess model assumptions. upper left: Residuals over predicted values. Upper right: Normal QQ plot of the residuals. Lower left: Square-root of the absolute values of the residuals versus fitted values. Lower right: Normal QQ plot of the random effect, birds In the Tukey-Anscombe plot we see that the residuals scatter around zero. Both QQ plots look fine. As there are no serious deviation from model assumptions (Nievergelt et al. 2015), we accepted this model.

**6.c) Drawing conclusions from model:**

#draw 2000 random values from the joint posterior distribution to obtain CrI
set.seed(0470) # specify the seed (starting value for your random generator)
nsim <- 2000
bsim.sp <- sim(mod0.sp, n.sim=nsim)
colnames(bsim.sp@fixef) <- names(fixef(mod0.sp)) # sim does not assign the names of the parameter in the current version.

#From the simulated values, the 2.5% and 97.5% quantiles can be used for the 95% credible interval
round(apply(bsim.sp@fixef, 2, quantile, prob=c(0.025,0.5,0.975)),2)

## (Intercept) log10(travel.sp) seasonspr
## 2.5% -0.35 -0.50 0.02
## 50% 1.68 0.20 0.12
## 97.5% 3.47 0.99 0.22
