# Supplementary material for: Flexible reaction norms to environmental variables along the migration route and the significance of stopover duration for total speed of migration in a songbird migrant
Source: Front Zool. 2017 Mar 20;14:17. doi: 10.1186/s12983-017-0203-3 (PMC5360013; doi:10.1186/s12983-017-0203-3)
Supplement: Additional file 5: — Migration routes of the Northern Wheatears, figures. (DOCX 239 kb) [file 12983_2017_203_MOESM5_ESM.docx]

Migration routes of the Northern Wheatears between their Alaskan breeding areas and African wintering grounds. The mean daily location estimates (autumn [before 1^st^ January]: blue filled circles, spring [after 1^st^ January]: orange filled triangles) and the 95% CrI of these location estimates (autumn: rosa, spring: yellow) are displayed. 24 hr daylight at high latitudes in spring did not yield location estimates. Birds were identified as in Table S1 in Additional file 2.

| **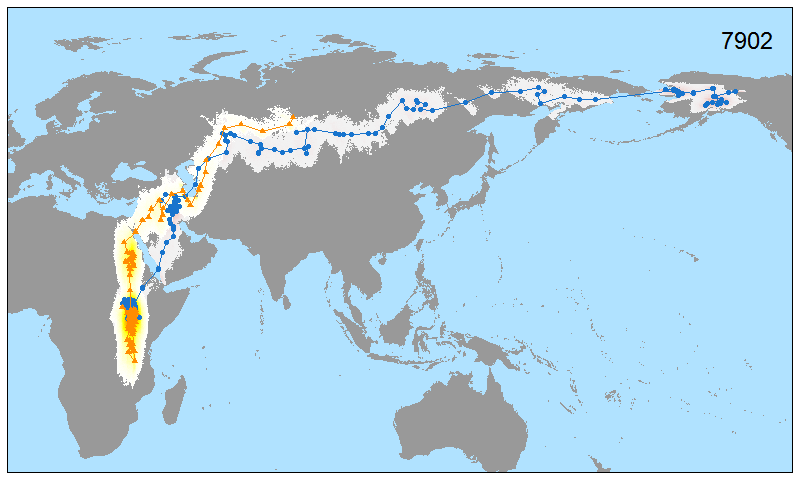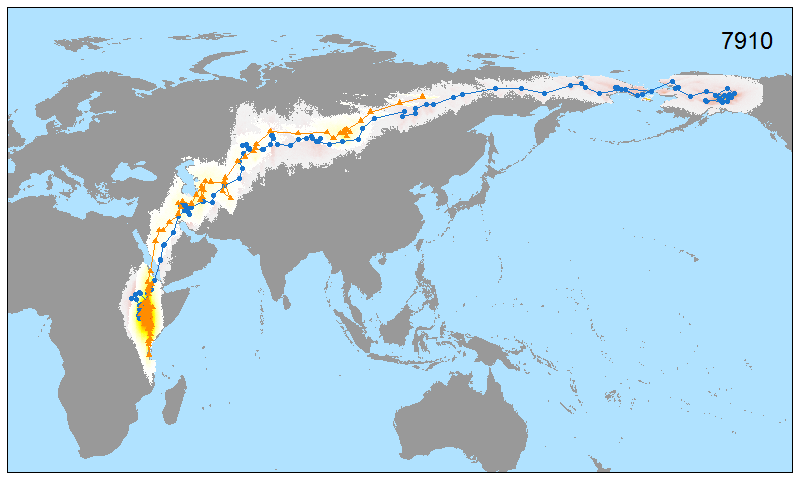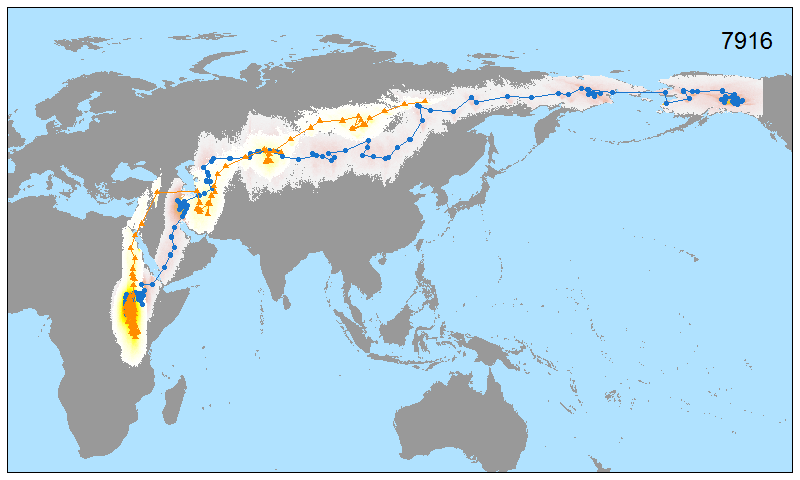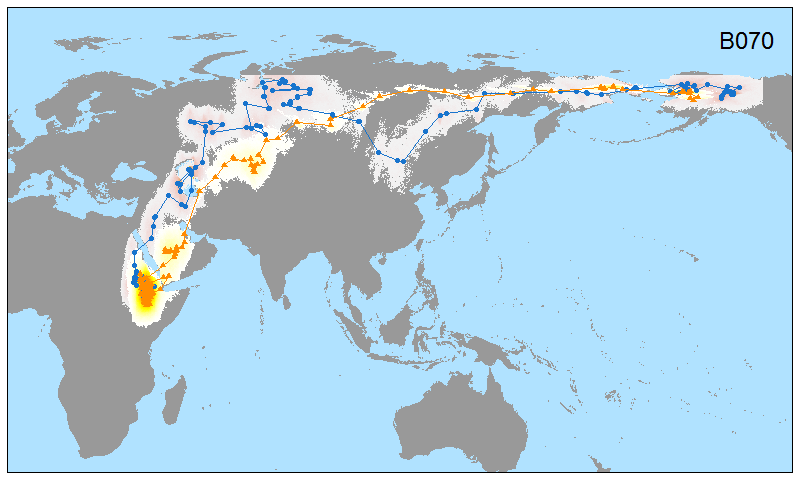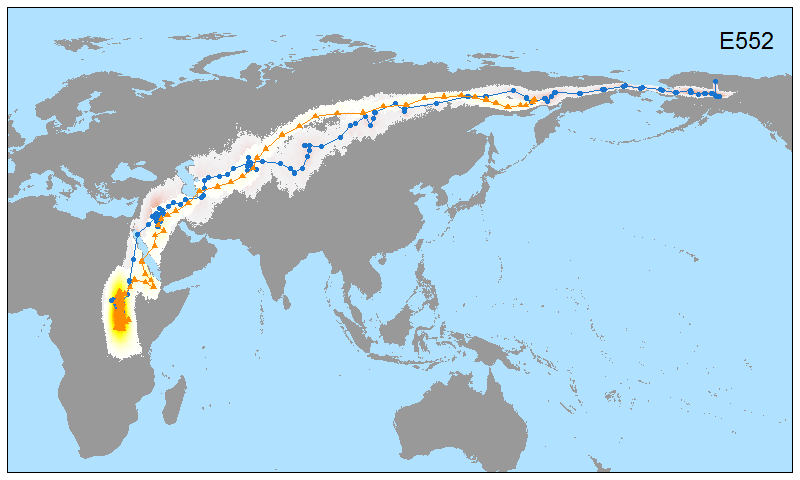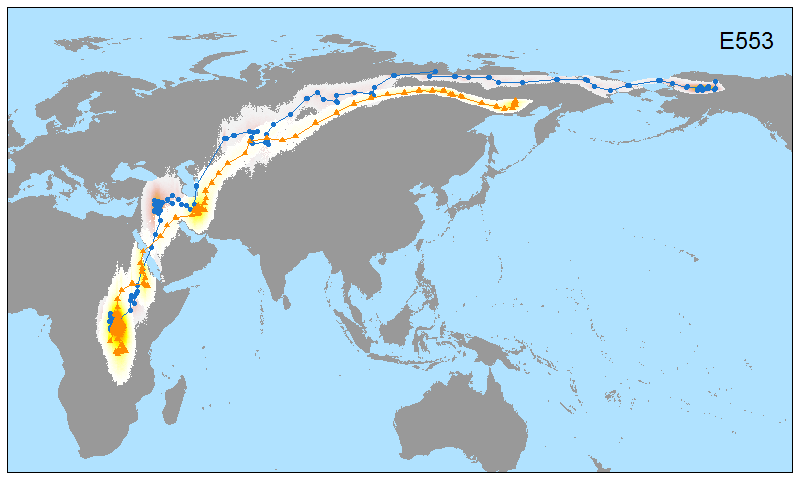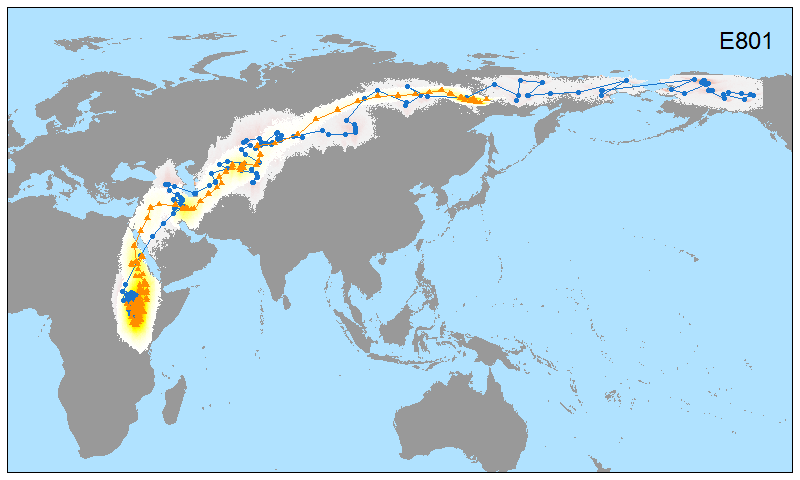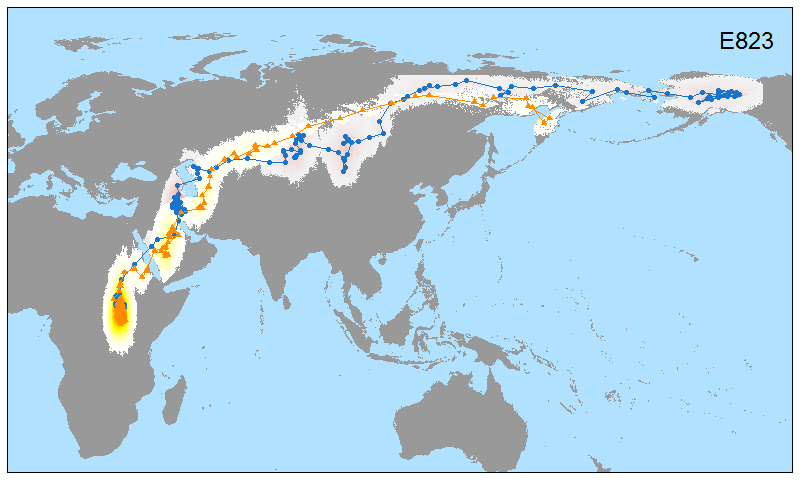** |
| --- |
