# Supplementary material for: Flexible reaction norms to environmental variables along the migration route and the significance of stopover duration for total speed of migration in a songbird migrant
Source: Front Zool. 2017 Mar 20;14:17. doi: 10.1186/s12983-017-0203-3 (PMC5360013; doi:10.1186/s12983-017-0203-3)

Environmental variables as experienced by northern wheatear during autumn migration 2009 and 2013 (upper four panels) and during spring migration 2010 and 2014 (lower panels).


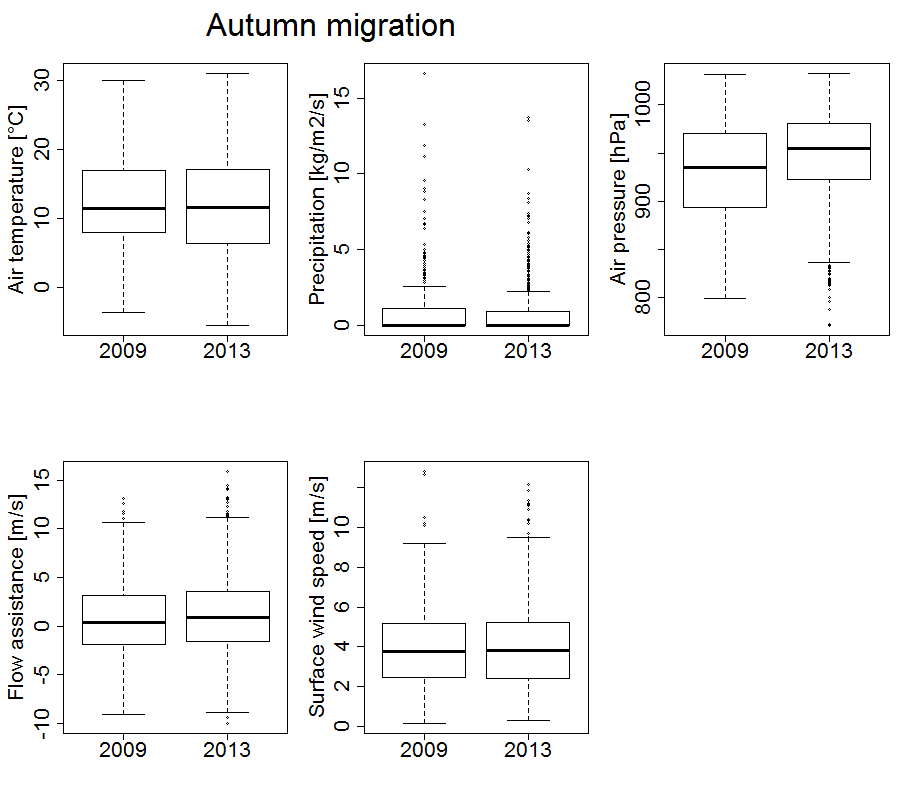


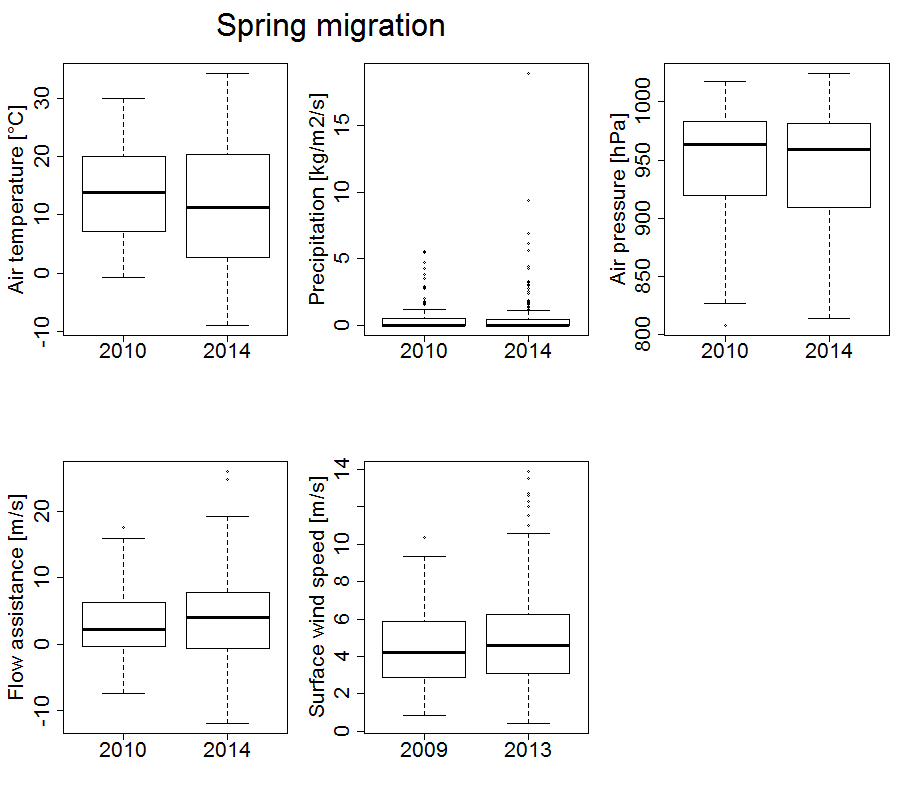

Supplement: Additional file 6: — Environmental conditions during autumn and spring migration, figures. (DOCX 108 kb) [file 12983_2017_203_MOESM6_ESM.docx]
