# Supplementary material for: Flexible reaction norms to environmental variables along the migration route and the significance of stopover duration for total speed of migration in a songbird migrant
Source: Front Zool. 2017 Mar 20;14:17. doi: 10.1186/s12983-017-0203-3 (PMC5360013; doi:10.1186/s12983-017-0203-3)

Experienced surface air temperature on each evening of migration at sunset over the remaining migration distance in autumn. Different shades of grey indicate surface air temperature as experienced by different birds of every single day during migration towards their East-African wintering grounds.


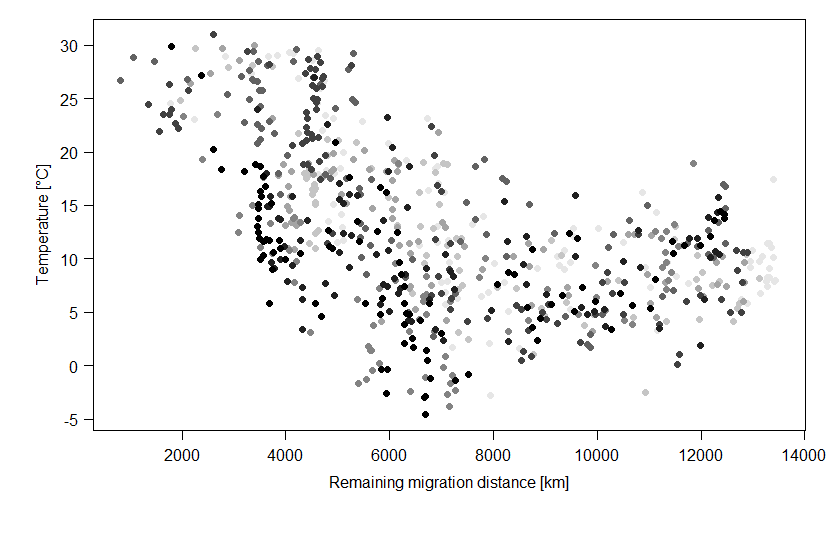

Supplement: Additional file 7: — Air temperature over remaining migration distance in autumn, figure. (DOCX 67 kb) [file 12983_2017_203_MOESM7_ESM.docx]
