# Supplementary material for: Flexible reaction norms to environmental variables along the migration route and the significance of stopover duration for total speed of migration in a songbird migrant
Source: Front Zool. 2017 Mar 20;14:17. doi: 10.1186/s12983-017-0203-3 (PMC5360013; doi:10.1186/s12983-017-0203-3)

Experienced (a) precipitation and (b) flow assistance at each evening at sunset of before performing a migratory flight in spring (n=153). Different shades of grey indicate environmental parameters as experienced by different birds of every single day when birds performed a migratory flight towards their East-African wintering grounds.

(a)


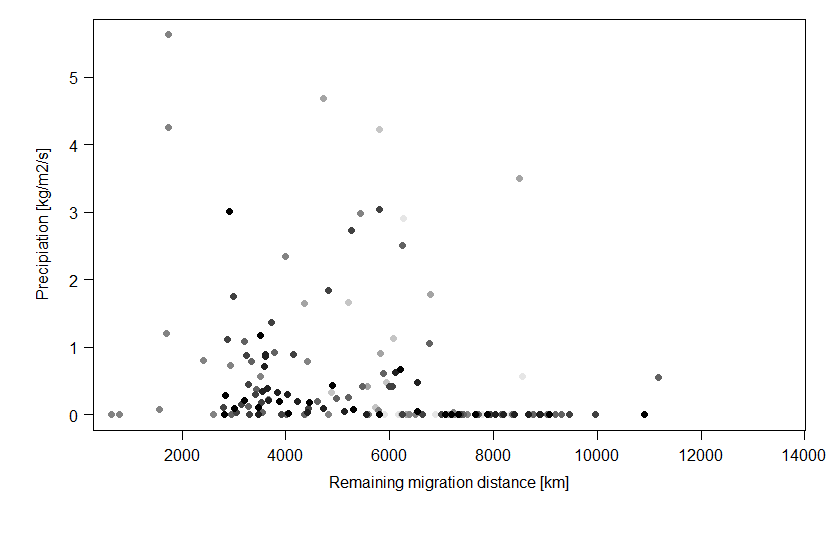


(b)


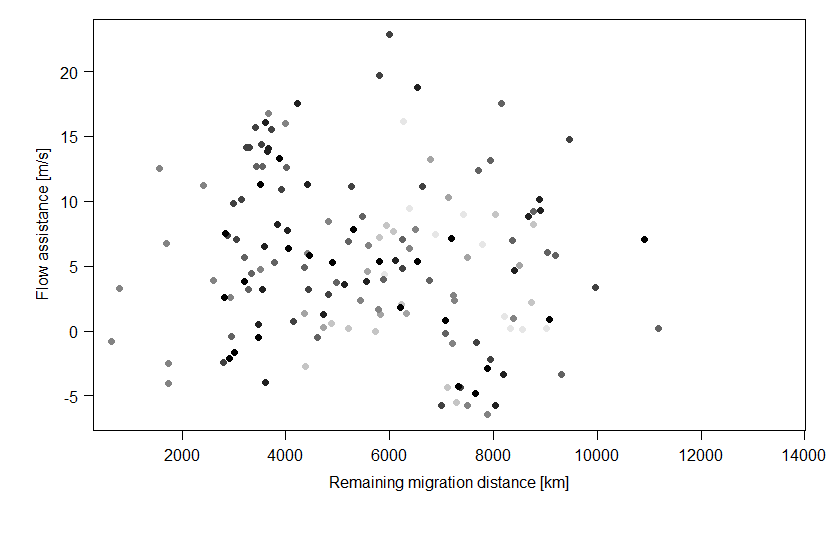

Supplement: Additional file 8: — Precipitation and flow assistance before performing a migratory flight in spring, figures. (DOCX 63 kb) [file 12983_2017_203_MOESM8_ESM.docx]
