# Supplementary material for: Flexible reaction norms to environmental variables along the migration route and the significance of stopover duration for total speed of migration in a songbird migrant
Source: Front Zool. 2017 Mar 20;14:17. doi: 10.1186/s12983-017-0203-3 (PMC5360013; doi:10.1186/s12983-017-0203-3)

Flow assistance to the next migratory site as described by modelled wind data for days when birds decided to stopover (“stopping”) and for days when birds decided to resume migration (“resuming migration”) during autumn migration. The latter included last day of stopovers and travel days.


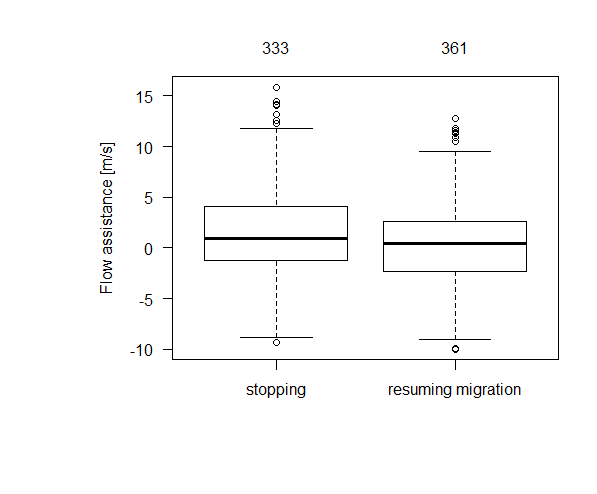

Supplement: Additional file 9: — Flow assistance during autumn migration, figure. (DOCX 47 kb) [file 12983_2017_203_MOESM9_ESM.docx]
